# Violence against healthcare professionals in intensive care units: a systematic review and meta-analysis of frequency, risk factors, interventions, and preventive measures

**DOI:** 10.1186/s13054-024-04844-z

**Published:** 2024-02-26

**Authors:** Sebastian Berger, Pascale Grzonka, Anja I. Frei, Sabina Hunziker, Sira M. Baumann, Simon A. Amacher, Caroline E. Gebhard, Raoul Sutter

**Affiliations:** 1 Clinic for Intensive Care, Department of Acute Medicine, University Hospital Basel, Petersgraben 4, 4031 Basel, Switzerland; 2 Medical Faculty, University of Basel, Basel, Switzerland; 3 Medical Communication and Psychosomatic Medicine, University Hospital Basel, Basel, Switzerland; 4 Department of Clinical Research, University of Basel, Basel, Switzerland

**Keywords:** Workplace violence, Intensive care units, Healthcare workers, Physical violence, Verbal violence

## Abstract

**Background:**

To assess the frequency, risk factors, consequences, and prevention of violence against healthcare workers in intensive care units.

**Methods:**

PubMed, Scopus, Google Scholar, EMBASE, Cochrane, and Web of Science were searched for studies on violence against healthcare workers in adult intensive care units. Risk factors, patient characteristics, and implications for healthcare workers were collected. Study quality, bias, and level of evidence were assessed using established tools.

**Results:**

Seventy-five studies with 139,533 healthcare workers from 32 countries were included. The overall median frequency of violence was 51% (IQR 37–75%). Up to 97% of healthcare workers experienced verbal violence, and up to 82% were victims of physical violence. Meta-analysis of frequency revealed an average frequency of 31% (95% CI 22–41%) for physical violence, 57% for verbal violence (95% CI 48–66%), and 12% for sexual violence (95% CI 4–23%). Heterogeneity was high according to the *I*^2^ statistics. Patients were the most common perpetrators (median 56%), followed by visitors (median 22%). Twenty-two studies reported increased risk ratios of up to 2.3 or odds ratios of up to 22.9 for healthcare workers in the ICU compared to other healthcare workers. Risk factors for experiencing violence included young age, less work experience, and being a nurse. Patients who exhibited violent behavior were often male, older, and physically impaired by drugs. Violence was underreported in up to 80% of cases and associated with higher burnout rates, increased anxiety, and higher turnover intentions. Overall the level of evidence was low.

**Conclusions:**

Workplace violence is frequent and underreported in intensive care units, with potential serious consequences for healthcare workers, calling for heightened awareness, screening, and preventive measures. The potential risk factors for violence should be further investigated.

*Systematic review registration*: The protocol for this review was registered with Prospero on January 15, 2023 (ID CRD42023388449).

**Supplementary Information:**

The online version contains supplementary material available at 10.1186/s13054-024-04844-z.

## Introduction

### Prevalence

Workplace violence (WPV) is defined by the US National Institute for Occupational Safety and Health as "violent acts including physical assaults and threats of assaults directed toward persons at work or on duty" [1, 2]. Data from the US Bureau of Labor Statistics and National Crime Victimization Survey show that healthcare workers (HCWs) experience a 20% higher rate of WPV compared to other employees [1, 3]. WPV could result in serious physical and psychological injury with long-term consequences for the affected HCWs such as sleep disorders, stress, increased turnover intention, and burnout [4, 5]. Violence against HCWs is not limited to North America as several studies indicate a rising incidence worldwide [6–9].

### Risk factors and potential consequences

Previous research suggests that violent acts against HCWs are severely underreported. This is due to several reasons, such as missing reporting systems or consequences [10–12]. Also, caregivers might interpret such incidents as a failure of their professional work, leading them to conceal experienced violent situations [11, 13, 14]. Although several reviews have addressed these issues, comprehensive data on the prevalence of violent acts against HCWs are mostly unsystematic and scarce [4, 7–9]. There is even less literature regarding the consequences of WPV for healthcare professionals, such as job dissatisfaction, burnout, other health-related outcomes, and increased turnover intentions [4, 5, 7, 15].

### Interventions and preventive measures

Few studies discussed the prevalence and risk factors of healthcare WPV with some proposing preventive measures [16, 17]. Systematic reviews of intensive care unit (ICU) cohorts are scarce and restricted to rural settings [18].

### Aims and significance of the study

The purpose of this study was to systematically assess the present literature regarding the prevalence, risk factors, interventions, and potential preventive measures for violence against HCWs in ICUs in order to allow more targeted efforts to combat workplace violence issues that lead to high job dissatisfaction and turnover intentions.

## Methods

### Search strategy

A search strategy was established using MeSH terms and keywords. These included "intensive care unit," "critical care," and "violence." The complete search strategy can be found in Additional file 1: Table S1.

The digital literature databases PubMed, EMBASE, the Cochrane database, SCOPUS, and Web of Science, as well as the search engine Google Scholar, were searched from their introduction until January 16, 2023. The protocol for this review was registered with Prospero on January 15, 2023 (ID CRD42023388449). ClinicalTrials.gov, the online registration platform, was searched for current studies involving violence in intensive care units. Non-English literature was translated using deepl.com. We imposed no time or language restrictions and included abstracts and conference posters. Furthermore, we screened the reference lists of the included studies for relevant references (citation tracking).

### Study selection, validation, and quality assessment

After removing duplicates, all titles and abstracts were screened by three reviewers (SB, PG, AF). In the next step, two independent reviewers (SB and RS) reviewed the full texts of the included papers. If no consensus was found, a third review was performed for final decision. The selection process was based on the following four pre-specified inclusion criteria: (1) adult patient population including (2) data regarding the frequency of violence and/or associated potential risks of WPV for HCWs in the ICU setting and/or (3) data regarding potential effects of violence, intervention targets, and/or preventive measures against violence in ICUs, and/or (4) data regarding underreporting of violent incidents. We excluded records not addressing violence against HCWs in ICUs, without peer review, and records not restricted to adult patients (i.e., age ≥ 18 years).

The validity of studies was assured by strictly adhering to the selection criteria mentioned above. Additionally, validity was assessed by quantifying the risk of bias and methodological quality of the included studies as follows: Two reviewers (SB and RS) used the Mixed Methods Appraisal Tool (MMAT) [19] for cross-sectional studies and the Newcastle Ottawa Quality Assessment Scale for observational studies [20] to assess the quality of the included studies and their risk of bias. For observational studies, selection was assessed by the representativeness of the included study population and the definition of violence used. Comparability was assessed by considering the studies controlling for risk factors and staff demographics. Exposure assessment criteria included the ascertainment of exposure and the non-response rate. For assessment of cross-sectional studies, similar criteria were applied such as the representativeness of the sample, the appropriate definition of violence, and the risk of non-response bias. The level of evidence was assessed using the OCEBM (Oxford Centre for Evidence Based Medicine) guidelines of the Levels of Evidence Working Group [21].

### Data extraction and reporting

Data from the suitable studies were extracted by one reviewer (SB) into a preformatted table, which was independently cross-checked by a second reviewer (RS). If available, the rates of verbal violence, physical violence, and sexual violence were assessed. We also extracted the frequency of incidents in which patients, relatives, or fellow HCWs, respectively, were the main perpetrators of these violent incidents. For studies that compared different frequencies of violence in different units (i.e., within or outside ICUs), we extracted the frequencies, odds, or risk of ICU staff being exposed to violence compared to other non-ICU wards of the hospital. We also extracted additional potential risk factors for the emergence of violence, which included patient characteristics and staff demographics. Information on the frequency of the violent acts being reported to supervisors, discovered by interviews, was extracted to display possible underreporting.

Finally, the potential effects of experiencing WPV, the implementation of interventions, as well as the introduction of preventive measures were collected.

The study conductance and reporting followed the PRISMA guidelines (Additional file 1: Table S2) [22].

### Statistical analysis

The data are presented qualitatively and quantitatively where appropriate. To visualize the studies’ geographic distribution, a world map was created on mapchart.net using a color scale to represent frequencies, with darker colors indicating more studies. A heatmap visualization tool was used to create a visual representation of the number of studies at each level of evidence in the following five areas: (1) the frequency of workers experiencing violent acts categorized into either physical violence, verbal violence, or sexual harassment, (2) associated potential risk factors, (3) underreporting, (4) potential consequences for healthcare personnel, and (5) reported interventions and preventive measures. The heatmap used a color scale to represent the number of studies, with darker colors representing more studies.

The frequency of verbal, physical, and sexual violence against HCWs medians and interquartile ranges (IQRs) was calculated when available. The frequency of HCWs being victims of violence was then compared by continents. Medians and IQRs of the frequency in which either patients, relatives, or fellow healthcare staff were the main perpetrators of this violence were also calculated. We then searched further for frequencies, odds, and risks of experiencing violence in the ICU when compared to other areas in the hospital such as emergency departments or general wards. However, the heterogeneity of these findings did not allow to conduct any further statistical analyses. Furthermore, the frequencies, odds, or relative risk for risk factors, such as patient characteristics or staff demographics, was extracted when available. The frequency of violent incidents not being reported as discovered by interviewing HCWs was also assessed, and medians and IQRs were calculated and compared between continents. Finally, data for potential consequences, interventions, and preventive measures were extracted. Meta-analysis of the frequency of violence and underreporting was performed using Stata/BE 18.0® (Stata Corp, 4095 Lakeway Drive, College Station, TX, USA) by computing the Freeman–Tukey double-arcsine-transformed proportion for each study and using a random effects model. Heterogeneity of studies was assessed using *I*^2^ statistic. Furthermore, we used the Louis Furuya-Kanamori (LFK) index to quantify asymmetry of small study effects [23].

## Results

**Fig. 1:**
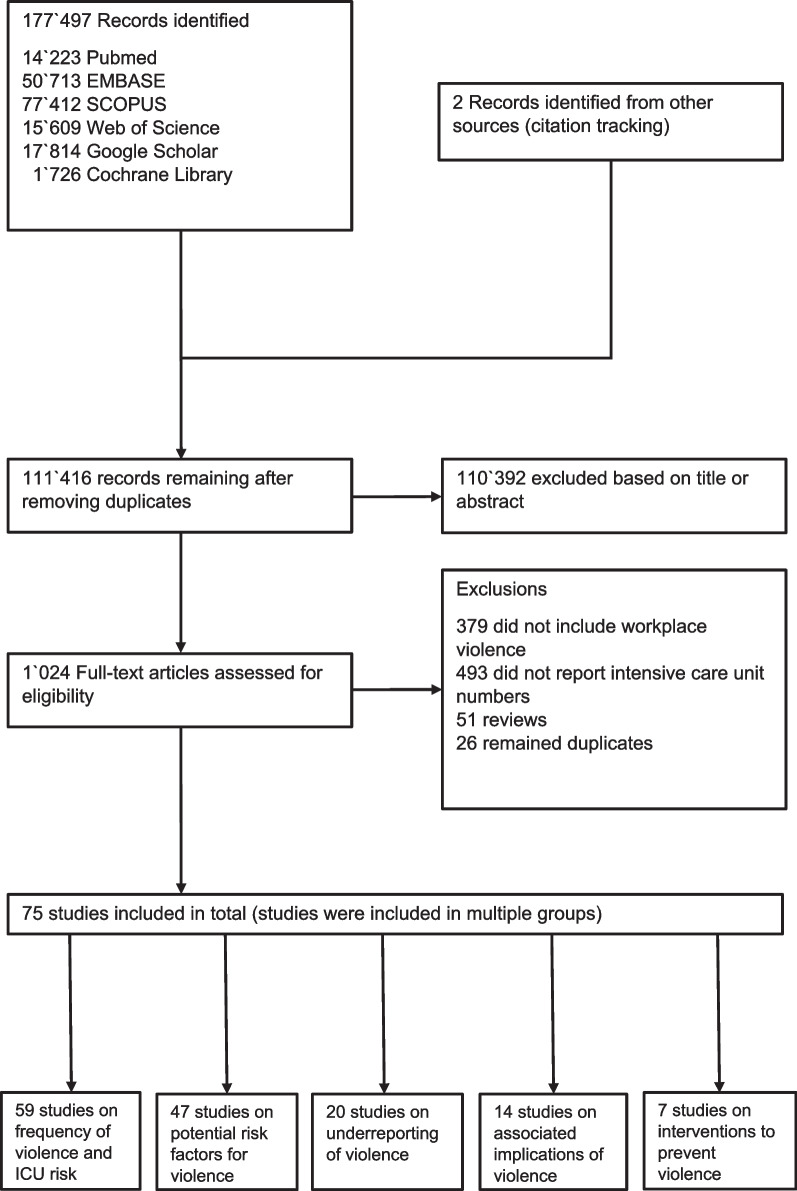
Study selection flow diagram

The study selection process is shown in Fig. 1. The initial search resulted in 177′497 references. After removing duplicates and screening the titles and abstracts, 1′024 references were included in the full text screening. Finally, 75 studies reporting a total of 139′533 HCWs from 32 countries were included (seven observational [17, 24–29] and 68 cross-sectional studies). The agreement coefficient κ between the two reviewers for the included studies was 0.93. For studies without agreements between the two reviewers, a third review was performed to reach consensus. Data acquisition was based on interviews or questionnaires [16, 30–96]. Fifty-one studies described the questionnaires [31–33, 36–44, 47–70, 72–80, 82, 91–94] with the majority using one of the following questionnaires (Workplace Violence Questionnaire [97] in 14 studies [31, 32, 36, 40, 50, 52, 54, 59, 64, 65, 67, 72, 76, 82], the Nursing Incivility Scale [98] in two studies [62, 96], and the Survey of Violence Experienced by Staff [99] in two studies [53, 70]).

**Fig. 2:**
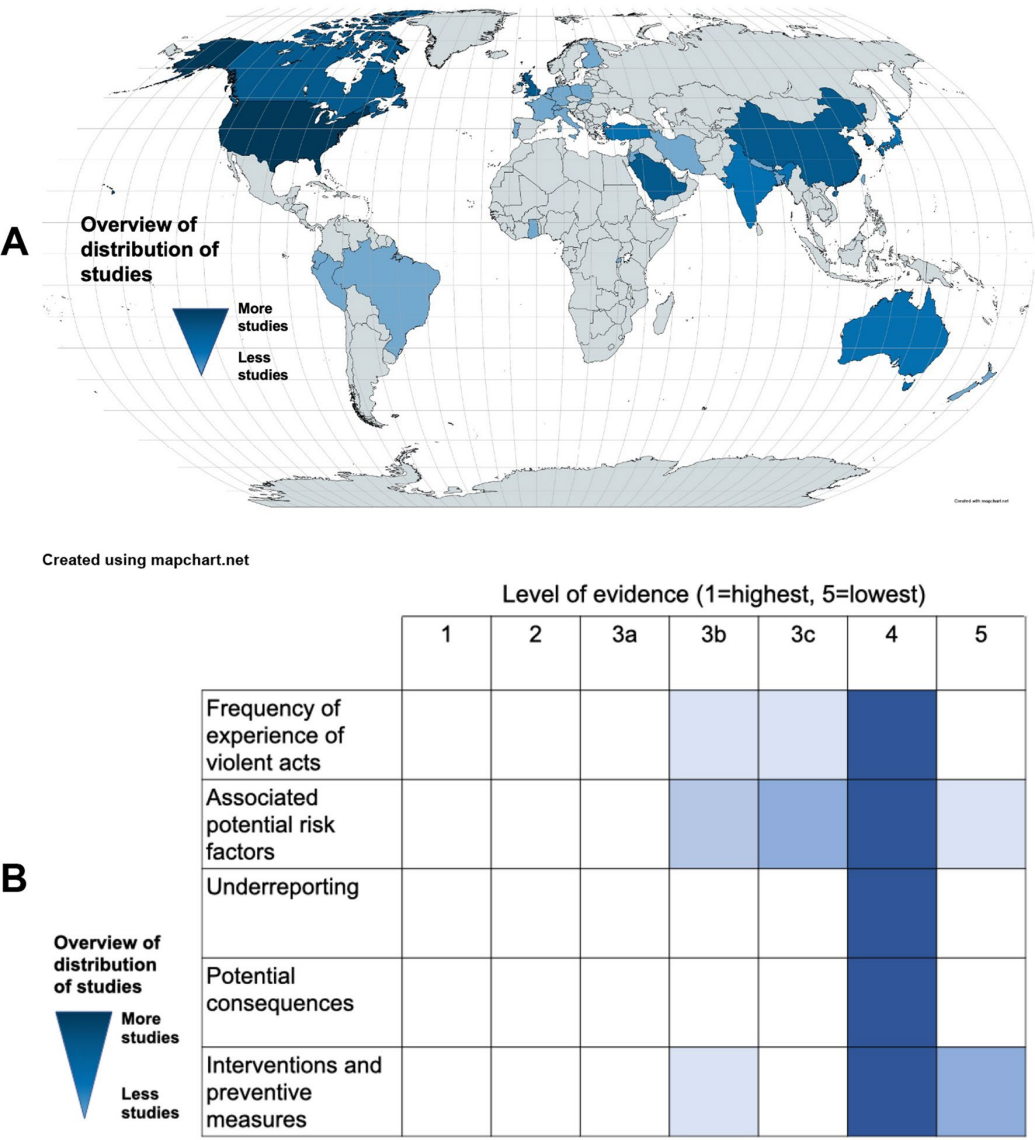
**A** Origin of studies and **B** level of evidence regarding specific characteristics

While 18 studies presented data strictly from 12′614 ICU personnel only [16, 17, 24, 35, 44, 46, 61, 63, 69, 71, 73, 74, 78, 79, 81, 82, 88, 90], 57 studies reported data from ICU HCWs as subgroups of larger heterogenic cohorts. Figure 1 presents the categorization of the included studies according to the reported findings. The main findings are presented in Table 1. Figure 2A presents the geographical distribution of included studies. No ongoing trials concerning WPV in ICUs were identified when searching ClinicalTrials.gov.

**Table 1:** Main findings from included studies

Origin Years of publication Number of studies	Frequency range of reported verbal violence	Frequency range of reported physical violence	Frequency range of reported sexual violence	Range of reported violent incidents with patients as main perpetrators	Range of reported violent incidents with relatives/family as main perpetrators	Reported violence in ICUs when compared to non-ICU wards
North America 2001–2022 (18 studies) [24, 25, 29, 37, 42, 45, 47, 49, 56, 57, 60, 62, 70, 71, 74, 87, 88, 90]	17–79%	7–60%	5–42%	35–98%	2–76%	RR 2.3 (95% CI 1–4)[25] OR 0.9 (95% CI 0.4–1.9)[37] to 4.9 (95% CI 2.6–9.2)[47]
South America 2018–2022 (3 studies) [44, 64, 83]	73–85%	0–25%	0–12%	53%^a^ [44]	18%^a^ [44]	OR 5.8 (95% CI 2–21)[64]
Europe 2002–2019 (12 studies) [28, 35, 38, 46, 51, 53, 54, 63, 66, 75, 89, 94]	38–97%	11–83%	68%^a^ [94]	35–87%	3–79%	OR 1.4 (95% CI 1–2)[53]
Africa 2017–2022 (2 studies) [36, 65]	55%	7–9%	2%^a^ [65]	NR	NR	No statistical difference
Asia 2012–2023 (36 studies) [27, 30–34, 39–41, 43, 48, 50, 52, 55, 58, 59, 61, 67, 68, 72, 73, 76–82, 84–86, 91–93, 95, 96]	19–90%	4–80%	3–23%	9–95%	6–82%	OR 0.2 (95% CI 0.0–1.1)[93] to 22.9 (95% CI 3–181)[52]
Australia & New Zealand 2019–2022 (4 studies) [16, 17, 26, 69]	57%^a^ [69]	NR	13%^a^ [69]	8–35%	0–2%	NR

^a^Only 1 study reporting (reference in parenthesis); NR = not reported

### Frequency of violence against HCWs in the ICU

**Fig. 3:**
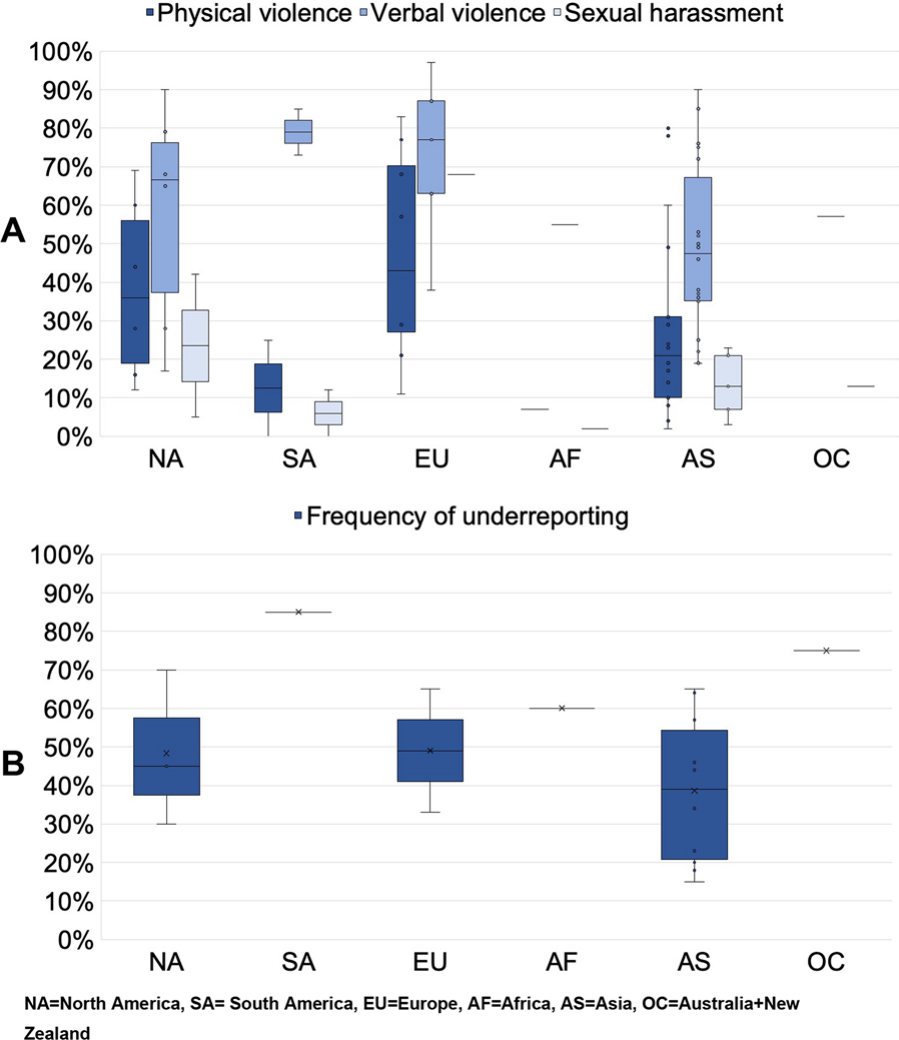
**A** Proportion of healthcare workers reporting experiences of violent incidents in ICUs of studies from different continents and **B** proportion of healthcare workers underreporting violent incidents as discovered by questionnaires (all expressed as median and interquartile ranges). NA = North America, SA = South America, EU = Europe, AF = Africa, AS = Asia, OC = Australia + New Zealand

The main findings of the 59 studies discussing frequencies, odds, or relative risks are presented in Table 1. The overall median frequency of any type of WPV, as reported in a total of 51 studies, was 54% (IQR 37–75%) [30–33, 35–50, 52–54, 56–61, 63–80, 82, 91, 93, 94]. Fifteen studies did not differentiate violence types, nor did they report explicit numbers from ICU personnel [31, 32, 36, 40, 42, 43, 45, 47, 49, 50, 53, 60, 67, 91, 93]. Thirty-six studies reported the frequency of HCWs who experienced physical violence, verbal violence, and sexual harassment or assault (Fig. 3A).

**Fig. 4:**
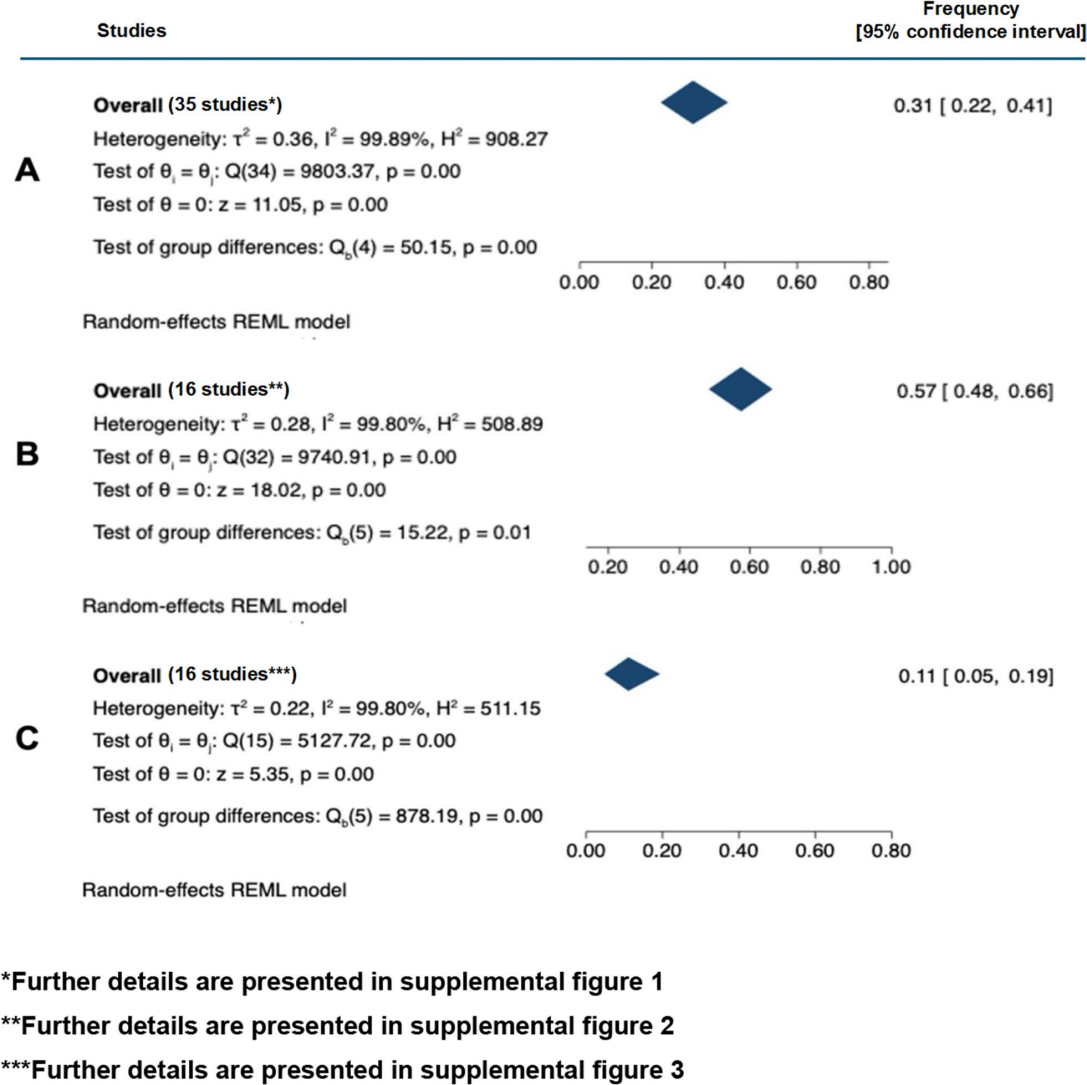
Meta-analysis of frequencies of (**A**) physical violence, (**B**) verbal violence, and (**C**) sexual violence in the included studies. *Further details are presented in Additional file 1: Fig. S1; **further details are presented in Additional file 1: Fig. S2; ***further details are presented in Additional file 1: Fig. S3

Physical violence was reported with a median proportion of 43% (IQR 27–70%) in eight European studies [35, 38, 46, 54, 63, 66, 75, 94], 36% (IQR 19–56%) in six North American studies [37, 56, 57, 70, 71, 74], and 21% (IQR 10–31%) in 18 Asian studies [30, 33, 39, 41, 48, 52, 58, 59, 61, 68, 72, 73, 76–78, 80, 82, 83]. Meta-analysis of this data revealed an average frequency of 31% (95% CI 22–41%). Heterogeneity of studies assessed by *I*^2^ statistics was high across all subgroups. The LFK index showed no asymmetry, suggesting low risk of publication bias (Fig. 4 and Additional file 1: Fig. S5).

Higher proportions of HCWs reported verbal violence with a median frequency of 77% (IQR 63–87%), compared to a median frequency of 67% (IQR 37–76%) and 48% (IQR 35–67%) for the above-mentioned North American and Asian studies, respectively. Meta-analysis of the included studies revealed an average frequency of 57% (95% CI 48–66%). There was, once again, high heterogeneity according to *I*^2^ statistics, and the LFK index showed minor asymmetry (Fig. 4 and Additional file 1: Fig. S5).

Sexual harassment was most commonly reported in North America (median of 24%; IQR 14–33%) [45, 56, 57], and 68% in a Slovakian study [94], followed by a median of 13% in Asian studies (IQR 7–21%) [30, 48, 68, 78, 80]. The average frequency of sexual violence was 12% (95% CI 4–23%) according to our meta-analysis with high heterogeneity according to *I*^2^ statistics and major asymmetry according to the LFK index (Fig. 4 and Additional file 1: Fig. S5). Four studies from South America, Africa and Australia, and New Zealand reported similar numbers for verbal, physical, and sexual violence (Table 1) [44, 64, 65, 69].

Details regarding the most common offenders can be found in Table 1. Visitors and relatives were the most common perpetrators of verbal violence in a median of 51% (IQR 29–71%) [31, 39, 41, 42, 45, 50, 56, 57, 59, 63, 67–70, 75, 79, 93], while they were the culprits in a median of 22% of physical violence events (IQR 9–55%) [31, 39, 41, 44, 56, 57, 59, 63, 66–68, 70, 75, 79]. Patients were the perpetrators in 40% of incidents of verbal violence (IQR 32–84%) [31, 39, 41, 42, 45, 50, 56, 57, 59, 63, 67–70, 75, 79, 93] and a median of 56% of physical violence incidents (IQR 33–89%) [31, 39, 41, 44, 56, 57, 59, 63, 66–68, 70, 75, 79]. Numbers regarding sexual violence were available from five cross-sectional studies with patients being the most frequent perpetrators (median frequency of 35%; IQR 35–59%), followed by coworkers and relatives (median 17%; IQR 6–22% and 8%; IQR 4–19%, respectively) [56, 57, 67–69].

### Comparison of WPV against HCWs between ICU and non-ICU wards

Forty-five studies analyzed differences between the frequency of violence against HCWs in ICUs and non-ICU wards (Table 1) [17, 25, 30–34, 36–43, 45, 47–60, 62, 64–68, 72, 75–77, 80, 91–94]. Six studies reported higher proportions, odds, or relative risks of ICU HCWs experiencing violence compared to HCWs of emergency departments (EDs) [17, 25, 62, 64, 72, 75]. Conversely, twelve studies reported lower frequencies, odds, or relative risks in ICUs compared to EDs (Table 1; details Additional file 1: Table S3) [30, 32–34, 37, 38, 43, 45, 51, 54, 66, 93]. When comparing ICUs to general wards, 16 studies reported higher frequencies, odds, or relative risks of violence for ICU staff compared to four studies reporting the opposite (Table 1; details Additional file 1: Table S3). The remaining seven cross-sectional studies reported similar frequencies, odds, or risks in the ICU and other units [36, 50, 55, 59, 65, 67, 91].

### Characteristics of HCWs and patients associated with WPV

Forty-seven studies reported on characteristics of HCWs, patients, and/or visitors associated with WPV (Additional file 1: Table S4). The most commonly described risks are outlined in Table 2, with younger age of the victim as the most prominent factor. Six cross-sectional studies from different countries reported decreasing odds (with ORs as low as 0.5) with increasing age or higher relative risks (as high as 1.3) to experience violence with decreasing age of workers [33, 39, 45, 53, 69, 77]. According to one American observational study, another factor was less work experience, revealing a relative risk of 1.4 (95% CI 0.9–2.1) for work experience of < 5 years as compared to > 15 years [25]. This finding was corroborated by four Chinese cross-sectional studies reporting two- to ninefold increased odds with a work experience of < 1 year compared to 5–15 years [59, 78, 80, 82].

**Table 2:** Most frequently reported potential risk factors for healthcare professionals and patient characteristics associated with experiencing violence

Risk factors of HCWs associated with experiencing violence from patients	Patient characteristics reported to be associated with violence against healthcare professionals
Younger Age [25, 31–33, 38, 39, 45, 53, 69, 72, 77, 81]	Impairment through drugs [17, 28, 47, 49, 53, 63, 70]
Low work experience [25, 50, 59, 78, 80, 82]	Miscommunication/Distrust [39, 50, 61, 72, 78, 82, 92]
Nursing profession [28, 33, 52, 60, 93]	Waiting hours/Inflexible visiting hours [39, 50, 61, 72, 78, 82, 92]
Shift work/Night shifts [61, 67, 78, 94]	Critical illness/delirium [17, 28, 47, 49, 53, 63, 70]
Understaffing/rural areas [36, 76]	Male and older patients [27, 49, 53]

There was no clear sex-related difference. While seven studies from Europe, India, and China reported male workers to experience violence more frequently [17, 38, 42, 43, 77, 80, 82], four studies found being female to be associated with higher odds of experiencing violence [31, 33, 39, 64].

Regarding profession, most data indicated that nurses were at higher risk for experiencing violence than doctors. One Chinese study reported nurses to be twice as likely to experience verbal violence compared to doctors. However, the same study showed the latter to have had a fivefold increased odds of experiencing physical violence [52]. Four further studies showed nurses to have higher frequencies of reported violence compared to physicians [28, 33, 60, 93].

Night shifts seem to be another potential risk factor, increasing the likelihood up to twofold to experience violence according to two cross-sectional studies from Nepal and China [67, 78].

The most frequently reported patient-related characteristic associated with violence was the impairment by illness or drugs [17, 28, 47, 49, 53, 63, 70] with illness severity attributed as the cause of 4% to 80% of violent episodes in two studies [28, 49]. Patients over the age of 65 years and male patients were reported to show violent behaviors more frequently, especially when being hospitalized for more than one week [26, 27, 49, 53]. Another factor causing violence, especially from families and relatives, was miscommunication, distrust against HCWs, and difficult patient situations or billing issues [39, 50, 61, 72, 78, 82, 92].

### Studies on potential underreporting of WPV

Twenty studies discussed potential underreporting [31–33, 36, 37, 41, 44, 45, 49, 50, 54, 61, 63, 66, 69, 70, 73, 78, 79, 82]. Eighteen of these used interviews to uncover failure to report violent events to supervisors [31–33, 36, 41, 44, 45, 49, 50, 61, 63, 66, 69, 70, 73, 78, 79, 82]. The frequencies of underreporting ranged from medians of 39 to 85% by continent and are shown in Fig. 3B (details Additional file 1: Table S5). Through meta-analysis, we reached an average frequency of underreporting of 47% (95% CI 37–58%) with high heterogeneity and a major asymmetry in the LFK index (Additional file 1: Figs. S4 and S5).

Several reasons were given for not reporting violent incidents. The most common was the feeling that nothing would change with reporting the assault [49, 50, 54, 61, 82], followed by the statement that violence was regarded as part of the job [49, 73, 82]. Notably, six cross-sectional studies revealed that no reporting systems were in place or that workers claimed to not have enough time or support to provide detailed reports [32, 37, 44, 49, 50, 63, 73].

#### Studies on potential consequences of WPV

Fourteen studies reported data on the effects of WPV on HCWs in ICUs [31, 34, 40, 42, 43, 49, 81, 83–87, 90, 95]. The most common are compiled in Table 3. Two studies reported violence to be a reason for work-related unhappiness and stress [83, 90]. Up to 13% of nurses reported persisting anxiety, sleep problems, and feelings of stress after experiencing violence, and over 22% changed their workplace due to experience of violence [49]. Associated symptoms were increased rates of headache, sleeping disturbances, tiredness, higher levels of anxiety, and negative effect on their everyday work including decreased productiveness [31, 42, 95]. This led to serious psychological problems, such as burnout [40]. Furthermore, nurses stated that incivility negatively impacted their patient safety competence [34, 84]. Three studies reported higher job dissatisfaction and an increased intention to leave the job due to violence [43, 86, 87].

**Table 3:** Most commonly reported potential consequences of experienced workplace violence for healthcare professionals

Potential negative effects of workplace violence on healthcare professionals	Potential negative effects of workplace violence on patients
Stress and burnout [31, 40, 43, 83, 85–87, 90] Increased rates of unhappiness and anxiety [31, 49, 81, 83, 90]	Less patient safety [34, 42, 84, 95] Worse nursing performance [31, 42, 95]
Increased turnover intention [31, 43, 49, 86, 87]	

Interventions to prevent workplace violence	Interventions to deal with workplace violence
Risk assessment tool for aggressive behavior [29, 89] No tolerance policy and visitor information [35] Increased patient safety culture [60]	De-escalation training [35] Enhanced security measures (security personnel, closed units) [88] Possible restrictive interventions and pharmacological sedation [89]

#### Interventions and preventive measures

Seven studies [24, 29, 35, 60, 71, 88, 89] discussed interventions to reduce or prevent WPV (Table 3). A study assessing the impact of a 60-min educational program for HCWs showed no difference regarding incidents before and after the education [24]. Another intervention encompassing training for definition of WPV, crisis prevention, therapeutic communication, and a risk assessment tool for aggressive behavior, as well as making an online reporting tool available, demonstrated an increase in staff confidence and ability to respond to WPV. However, the frequency of events did not change [29]. In a cross-sectional study from 2022, a higher level of patient safety culture was associated with 0.5 times lower odds of WPV and burnout scores among staff [60]. Other measures reported to help combat WPV were conflict resolution training for all staff, displaying posters which outline a no violence tolerance, using the Broset checklist [100], de-escalation strategies, considering restrictive interventions or pharmacological sedation, and personal alarms for areas of lone working [35, 89]. However, further data regarding the effects of such measures could not be identified.

#### Risk of bias, quality of studies, and level of evidence

The quality assessment (Additional file 1: Table S6) revealed a heterogeneous quality of the included cohort studies (three high-quality studies [17, 25, 26] and four fair or even poor-quality studies [24, 27–29]). There was a high risk of selection bias, lack of comparability of cohorts, and short follow-up time [24, 29]. The quality assessment of the included cross-sectional studies (Additional file 1: Table S7) revealed an overall high risk of recall bias. There was also an overall high risk of reporting bias, with 47 studies having high non-response rates of over 20% [16, 30, 33–38, 41, 44, 45, 47–51, 53, 56, 57, 59, 61–63, 66, 68, 70, 71, 74–80, 82–90, 92, 93, 96]. Furthermore, the cross-sectional nature did not permit any causal conclusions. Generally, the level of evidence in the included studies was low (Fig. 2B).

## Discussion

Our systematic review found WPV to be a common but under-investigated problem in ICU. Furthermore, WPV seems to occur more frequently in ICUs than on other wards, despite a presumable high number of unreported cases. Verbal violence was the most common type of WPV, with some studies reporting an almost 100% exposure of HCWs. The perpetrators were mostly patients, their relatives, and other visitors. There were multiple studies showing high rates of physical violence affecting up to 80% of HCWs. The assaults ranged from scratching and biting to hitting and inflicting physical damage. Violent behavior was most commonly exhibited by patients or visitors. Another severe form of aggression toward HCWs was sexual harassment and assaults. Here, studies showed that ICU practitioners are also at risk of experiencing such violence also from coworkers or superiors [44, 57], but patients were still the most frequent offenders [68]. The frequency of physical violence in ICUs was substantial (up to 83%), and the high proportion of HCWs not reporting such violence in the initial phase suggests that physical violence is even higher than reported by the World Health Organization (WHO) in 2022 with an estimated 8% to 38% of HCWs experiencing physical violence [101]. Our meta-analysis of frequencies found an overall frequency of 31% for physical violence. The meta-analysis of verbal violence showed an even higher average frequency of 57%, while sexual violence was still considerably frequent with an average of 11%. The high heterogeneity to these findings and some important asymmetry of the study effect sizes according to the LFK index have to however be taken into consideration when interpreting these study results. Lack of uniform questionnaires and definitions for violence challenge comparisons between reported frequencies. Unfortunately, the evidence provided by the included studies was low and subject to a high risk of bias.

Furthermore, many studies had high non-response rates. Therefore, it is possible that HCWs who experienced violence in the past were more likely to refrain from study participation. Secondly, since violent events are severely traumatic for HCWs, there might be a recall bias possibly leading healthcare professionals to overestimate the rate of violent events. We aimed to directly compare the risk of HCWs in ICUs to known high-risk units, such as EDs and to general units across the hospital. Here we revealed widely differing results when it comes to frequencies, odds, or relative risk of violence in the ICU compared to EDs. Based on several work-related similarities in EDs and ICUs (shift work, management of critically ill patients, or emergency scenarios), it seems plausible that the risk of ICU workers for WPV is at least similar to that of the ED HCWs. When comparing the risk of WPV in the ICU and on general wards, there were considerably more studies revealing increased frequencies, odds, or relative risks in the ICU. Regional differences were considerable (as illustrated in Fig. 3A), explained in part by the different patient populations, cultural differences, and varying resources to treat patients and report violent events.

Further risk factors associated with exposure to violence were younger age and less work experience. In addition, nurses seemed to experience more violence in comparison with physicians, but results were conflicting. Shift and night work was found to be an additional risk factor, probably because of the reduced number of staff overnight. This may in part explain why HCWs in the ICU or the ED are at higher risk of experiencing violence.

As to those exerting violence, patients that were older and male were more likely to exhibit such behavior. Violence from visitors and families was more common if there was miscommunication or distrust against the doctors, especially if the patient was severely ill or died, adding another mechanistic hypothesis for increased experience of WPV in ICUs. Illness and delirium were attributed to be the cause of up to 80% of violent incidents committed by patients [28, 49]. This may explain why a lot of healthcare staff, especially in the ICU, did not report violent incidents, as they regarded them as a part of the job or even felt the expression of violence was due to a personal failure to attend to the patient's requirements.

**Fig. 5:**
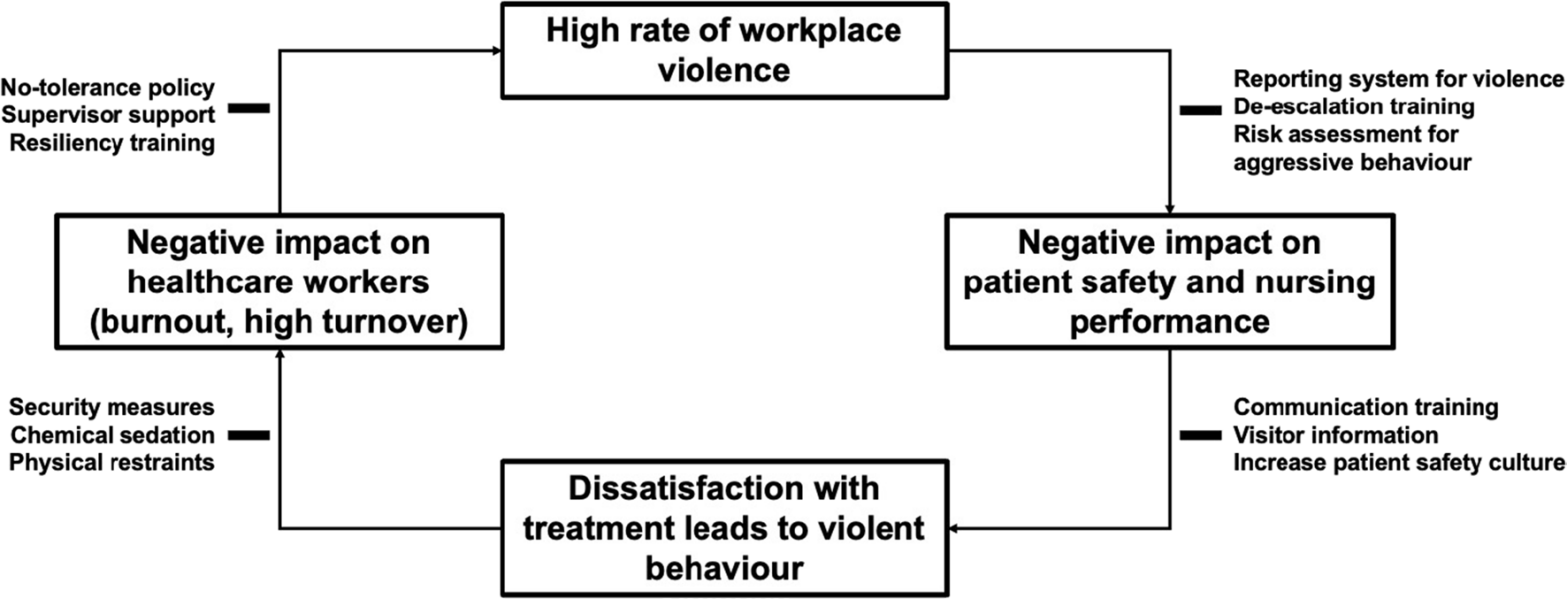
Vicious cycle of workplace violence and possible intervention targets and preventive measures

Our review additionally found that the rate of not reporting violent incidents, as discovered by interviewing healthcare staff, varied substantially. Besides the belief that WPV is regarded as part of the job, another explanation may be that chemical sedation and physical restraints are more readily available in the ICU. Therefore HCWs might use these tools early before calling for help. Other suggested reasons for underreporting were lack of support from superiors, lack of reporting systems, and a disillusionment of HCWs with the healthcare system, as many workers assumed that reporting incidents would not result in any positive change. This disillusionment might stem from the negative impact WPV has on HCWs as uncovered in 14 studies, including job dissatisfaction, burnout, increased rates of unhappiness, stress, anxiety, other health-related outcomes, and higher turnover intentions. The latter might worsen the global staff shortage [102, 103] which has already been amplified by the recent COVID-19 pandemic [104, 105]. Together with understaffing, also reported to increase the risk of WPV [37], the latter might lead to a vicious circle (Fig. 5). These potential negative effects underscore the importance of more rigorous screening, prevention, and handling of WPV in ICUs.

We present possible interventions to combat WPV as suggested by the included studies (Fig. 5) and suggest their implementation to prevent WPV. Among the reported interventions were different trainings for healthcare staff in de-escalation techniques and communication courses aimed to prevent miscommunication and to address the patients’ and relatives’ dissatisfaction with treatment, as frustration and resentments can trigger aggression toward HCWs. Furthermore, the importance of reporting systems and risk assessments for aggressive behavior was highlighted in different studies [29, 32, 37, 44, 49, 50, 63, 73, 89]. To prevent HCWs from leaving the profession, there should be increased supervisor support, resiliency training, and clear communication of a no-tolerance policy against violence. Finally, to prevent negative impact and physical harm being caused by WPV, it is important for hospitals to invest in security measures. To deal with patients exhibiting violent behaviors, it is important to have protocols in place to perform standardized measures including chemical sedation, use of physical restraints, and pharmacological and non-pharmacological treatments to prevent delirium [106], since delirious patients tended to be more violent [27, 70].

With the high risk of bias there might be considerable confounding in the reporting of proportions of HCWs experiencing violence and potential risk factors. On the other hand, underreporting still seems to be a substantial issue, possibly leading to an underestimation of WPV in ICUs and the healthcare system in general. These concerns are further amplified by the lack of ongoing trials on WPV in ICUs, even in times with growing shortage of healthcare personnel following the recent pandemic.

## Conclusion

Our review identified several heterogeneous studies conducted worldwide reporting violence against HCWs in the ICU to be frequent but underreported, with potential serious consequences. WPV in ICU workers was more frequent when compared to workers in general wards but similar when compared to ED personnel. Younger and less experienced HCWs seem to be more susceptible to WPV, while older and male patients suffering from severe illness, delirium, and dementia are more likely to exhibit violent behavior. These factors should be further investigated and validated for use in screening tools. The experience of WPV is associated with higher burnout rates, increased anxiety, and higher turnover intentions. Despite such severe consequences, we found up to 80% of incidents not being reported initially. Often reporting systems were not in place or there was a disillusionment with the healthcare system and its ability for change. The available evidence is subject to a significant risk of bias and confounding. While awareness for WPV seems high at first glance, it is important that measures are put into place to combat the growing epidemic of WPV. Thus, we summarized and suggested intervention targets for prevention and dealing with violence in ICUs and healthcare in general. The potential risk factors for violence against HCWs in ICUs described above should be further investigated and validated for use in screening tools. Further randomized prospective trials are urgently needed to improve reporting of these incidents and to gain a better understanding of the effectiveness of screening and preventive measures.

### Supplementary Information

**Additional file 1: Supplementary Table 1**. Search documentation including all of the keywords and MeSH terms used. **Supplementary Table 2**. Prisma checklist.** Supplementary Table 3**. Reported frequency and risk of workplace violence encountered by HCWs in ICUs.** Supplementary Table 4**. Staff demographics and patient characteristics associated with workplace violence.** Supplementary Table 5**. Frequency of underreporting violent incidents as discovered by interviews with healthcare staff and reasions for underreporting.** Supplementary Table 6**. Assessment of included cohort studies according to the Newcastle-Ottawa Quality Assessment Form.** Supplementary Table 7**. Assessment of included cross-sectional studies according to the mixed methods appraisal tool (MMAT).** Supplementary Figure 1**. Meta-analysis of frequency of physical violence. **Supplementary Figure 2.** Meta-analysis of frequency of verbal violence.** Supplementary Figure 3**. Meta-analysis of sexual violence.** Supplementary Figure 4**. Meta-analysis of frequency of underreporting of violent events. **Supplementary Figure 5**. LFK index for asymmetry for meta-analysis of (**A**) physical violence, (**B**) verbal violence, (**C**) sexual violence and (**D**) underreporting of violent events.

## Data Availability

All data generated or analyzed during this study are included in this published article and its supplementary information files.
