# Supplementary material for: Violence against healthcare professionals in intensive care units: a systematic review and meta-analysis of frequency, risk factors, interventions, and preventive measures
Source: Crit Care. 2024 Feb 26;28:61. doi: 10.1186/s13054-024-04844-z (PMC10898135; doi:10.1186/s13054-024-04844-z)
Supplement: Supplementary file 1 — Additional file 1: Supplementary Table 1. Search documentation including all of the keywords and MeSH terms used. Supplementary Table 2. Prisma checklist. Supplementary Table 3. Reported frequency and risk of workplace violence encountered by HCWs in ICUs. Supplementary Table 4. Staff demographics and patient characteristics associated with workplace violence. Supplementary Table 5. Frequency of underreporting violent incidents as discovered by interviews with healthcare staff and reasions for underreporting. Supplementary Table 6. Assessment of included cohort studies according to the Newcastle-Ottawa Quality Assessment Form. Supplementary Table 7. Assessment of included cross-sectional studies according to the mixed methods appraisal tool (MMAT). Supplementary Figure 1. Meta-analysis of frequency of physical violence. Supplementary Figure 2. Meta-analysis of frequency of verbal violence. Supplementary Figure 3. Meta-analysis of sexual violence. Supplementary Figure 4. Meta-analysis of frequency of underreporting of violent events. Supplementary Figure 5. LFK index for asymmetry for meta-analysis of (A) physical violence, (B) verbal violence, (C) sexual violence and (D) underreporting of violent events. [file 13054_2024_4844_MOESM1_ESM.docx]

**Supplemental Table 1: Search Documentation**

Searched on January 16, 2023

| Concept 1  **Intensive Care Unit** | MeSH:  "intensive care units"[MeSH Terms]  OR  MeSH:  "critical care"[MeSH Terms]  OR  Keywords:  „intensive care unit“[Title/Abstract] OR „intensive care“[Title/Abstract] OR „critical care“[Title/Abstract] OR „critical care staff“[Title/Abstract] OR „critical care nurse“[Title/Abstract] OR „intensive care staff“[Title/Abstract] OR „intensive care nurse“[Title/Abstract] OR „critical care nursing“[Title/Abstract] OR nurse*[Title/Abstract] OR doctor*[Title/Abstract] OR ICU[Title/Abstract] OR CCU[Title/Abstract] OR SICU[Title/Abstract] OR MICU[Title/Abstract] |
| --- | --- |

| Concept 2  **Violence** | MeSH:  "Violence"[MeSH Terms]  OR  Keywords:  violence[Title/Abstract] OR „workplace violence“[Title/Abstract] OR violent[Title/Abstract] OR „violent patients“[Title/Abstract] OR abuse[Title/Abstract] OR „verbal abuse“[Title/Abstract] OR „physical abuse“[Title/Abstract] OR „exposure to violence“[Title/Abstract] OR „psychological violence“[Title/Abstract] OR „sexual harassment“[Title/Abstract] OR „non-physical violence“[Title/Abstract] OR attack*[Title/Abstract] |
| --- | --- |

**PUBMED**

("intensive care units"[MeSH Terms] OR "critical care"[MeSH Terms] OR „intensive care unit“[Title/Abstract] OR „intensive care“[Title/Abstract] OR „critical care“[Title/Abstract] OR „critical care staff“[Title/Abstract] OR „critical care nurse“[Title/Abstract] OR „intensive care staff“[Title/Abstract] OR „intensive care nurse“[Title/Abstract] OR „critical care nursing“[Title/Abstract] OR nurse*[Title/Abstract] OR doctor*[Title/Abstract] OR ICU[Title/Abstract] OR CCU[Title/Abstract] OR SICU[Title/Abstract] OR MICU[Title/Abstract]) AND ("Violence"[MeSH Terms] OR violence[Title/Abstract] OR „workplace violence“[Title/Abstract] OR violent[Title/Abstract] OR „violent patients“[Title/Abstract] OR abuse[Title/Abstract] OR „verbal abuse“[Title/Abstract] OR „physical abuse“[Title/Abstract] OR „exposure to violence“[Title/Abstract] OR „psychological violence“[Title/Abstract] OR „sexual harassment“[Title/Abstract] OR „non-physical violence“[Title/Abstract] OR attack*[Title/Abstract])

**EMBASE**

('intensive care unit'/exp OR 'intensive care'/exp OR 'intensive care unit' OR 'intensive care' OR 'critical care' OR 'critical care staff' OR 'critical care nurse' OR 'intensive care staff' OR 'intensive care nurse' OR 'critical care nursing' OR nurse* OR doctor* OR ICU OR CCU OR SICU OR MICU) AND ('violence'/exp OR 'violence' OR 'workplace violence' OR violent OR 'violent patients' OR abuse OR 'verbal abuse' OR 'physical abuse' OR 'exposure to violence' OR 'psychological violence' OR 'sexual harassment' OR 'non-physicial violence' OR attack*)

**SCOPUS**

( "intensive care unit" OR "intensive care" OR "critical care" OR TITLE-ABS-KEY ( "critical care staff" ) OR TITLE-ABS-KEY ( "critical care nurse" ) OR TITLE-ABS-KEY ( "intensive care staff" ) OR TITLE-ABS-KEY ( "intensive care nurse" ) OR TITLE-ABS-KEY ( "critical care nursing" ) OR TITLE-ABS-KEY ( nurse* ) OR TITLE-ABS-KEY ( doctor* ) OR TITLE-ABS-KEY ( icu ) OR TITLE-ABS-KEY ( ccu ) OR TITLE-ABS-KEY ( sicu ) OR TITLE-ABS-KEY ( micu ) ) AND ( violence OR TITLE-ABS-KEY ( violence ) OR TITLE-ABS-KEY ( "workplace violence" ) OR TITLE-ABS-KEY ( violent ) OR TITLE-ABS-KEY ( "violent patients" ) OR TITLE-ABS-KEY ( abuse ) OR TITLE-ABS-KEY ( "verbal abuse" ) OR TITLE-ABS-KEY ( "physical abuse" ) OR TITLE-ABS-KEY ( "exposure to violence" ) OR TITLE-ABS-KEY ( "psychological violence" ) OR TITLE-ABS-KEY ( "sexual harassment" ) OR TITLE-ABS-KEY ( "non-physicial violence" ) OR TITLE-ABS-KEY ( attack* ) )

**Web of Science**

(ALL=(intensive care unit) OR ALL=(intensive care) OR ALL=(critical care) OR AB=(critical care staff) OR AB=(critical care nurse) OR AB=(intensive care staff) OR AB=(intensive care nurse) OR AB=(critical care nursing) OR AB=(nurse*) OR AB=(doctor*) OR AB=(icu) OR AB=(ccu) OR AB=(micu) OR AB=(sicu) OR TI=(critical care staff) OR TI=(critical care nurse) OR TI=(intensive care staff) OR TI=(intensive care nurse) OR TI=(critical care nursing) OR TI=(nurse*) OR TI=(doctor*) OR TI=(icu) OR TI=(ccu) OR TI=(micu) OR TI=(sicu) OR AK=(critical care staff) OR AK=(critical care nurse) OR AK=(intensive care staff) OR AK=(intensive care nurse) OR AK=(critical care nursing) OR AK=(nurse*) OR AK=(doctor*) OR AK=(icu) OR AK=(ccu) OR AK=(micu) OR AK=(sicu)) AND (ALL=(violence) OR ALL=(workplace violence) OR AB=(violent) OR AB=(violent patients) OR AB=(abuse) OR AB=(verbal abuse) OR AB=(physical abuse) OR AB=(exposure to violence) OR AB=(psychological violence) OR AB=(sexual harassment) OR AB=(non-physical violence) OR AB=(attack*) OR TI=(violent) OR TI=(violent patients) OR TI=(abuse) OR TI=(verbal abuse) OR TI=(physical abuse) OR TI=(exposure to violence) OR TI=(psychological violence) OR TI=(sexual harassment) OR TI=(non-physical violence) OR TI=(attack*) OR AK=(violent) OR AK=(violent patients) OR AK=(abuse) OR AK=(verbal abuse) OR AK=(physical abuse) OR AK=(exposure to violence) OR AK=(psychological violence) OR AK=(sexual harassment) OR AK=(non-physical violence) OR AK=(attack*))

**Google Scholar**

(“intensive care units” OR “critical care” OR „intensive care unit“ OR „intensive care“ OR „critical care“ OR „critical care staff“ OR „critical care nurse“ OR „intensive care staff“ OR „intensive care nurse“ OR „critical care nursing“ OR nurse* OR doctor* OR ICU OR CCU OR SICU OR MICU) AND (“Violence” OR violence OR „workplace violence“ OR violent OR „violent patients“ OR abuse OR „verbal abuse“ OR „physical abuse“ OR „exposure to violence” OR „psychological violence“ OR „sexual harassment“ OR „non-physical violence“ OR attack*)

**Cochrane Library**

1726 Cochrane Reviews matching ("intensive care units" OR "critical care" OR „intensive care unit“ OR „intensive care“ OR „critical care“ OR „critical care staff“ OR „critical care nurse“ OR „intensive care staff“ OR „intensive care nurse“ OR „critical care nursing“ OR nurse* OR doctor* OR ICU OR CCU OR SICU OR MICU) AND ("Violence" OR violence OR „workplace violence“ OR violent OR „violent patients“ OR abuse OR „verbal abuse“ OR „physical abuse“ OR „exposure to violence” OR „psychological violence“ OR „sexual harassment“ OR „non-physical violence“ OR attack*) in All Text

**Supplemental Table 2: Prisma checklist**

| **Section and Topic** | **Item #** | **Checklist item** | **Location where item is reported** |
| --- | --- | --- | --- |
| **TITLE** | | |  |
| Title | 1 | Identify the report as a systematic review. | Page 1 |
| **ABSTRACT** | | |  |
| Abstract | 2 | See the PRISMA 2020 for Abstracts checklist. | Page 2 |
| **INTRODUCTION** | | |  |
| Rationale | 3 | Describe the rationale for the review in the context of existing knowledge. | Page 4 |
| Objectives | 4 | Provide an explicit statement of the objective(s) or question(s) the review addresses. | Page 4 |
| **METHODS** | | |  |
| Eligibility criteria | 5 | Specify the inclusion and exclusion criteria for the review and how studies were grouped for the syntheses. | Page 5/6 |
| Information sources | 6 | Specify all databases, registers, websites, organisations, reference lists and other sources searched or consulted to identify studies. Specify the date when each source was last searched or consulted. | Page 5/6 |
| Search strategy | 7 | Present the full search strategies for all databases, registers and websites, including any filters and limits used. | Page 5/6 |
| Selection process | 8 | Specify the methods used to decide whether a study met the inclusion criteria of the review, including how many reviewers screened each record and each report retrieved, whether they worked independently, and if applicable, details of automation tools used in the process. | Page 5/6 |
| Data collection process | 9 | Specify the methods used to collect data from reports, including how many reviewers collected data from each report, whether they worked independently, any processes for obtaining or confirming data from study investigators, and if applicable, details of automation tools used in the process. | Page 5/6 |
| Data items | 10a | List and define all outcomes for which data were sought. Specify whether all results that were compatible with each outcome domain in each study were sought (e.g. for all measures, time points, analyses), and if not, the methods used to decide which results to collect. | Page 5/6 |
|  | 10b | List and define all other variables for which data were sought (e.g. participant and intervention characteristics, funding sources). Describe any assumptions made about any missing or unclear information. | Page 5/6 |
| Study risk of bias assessment | 11 | Specify the methods used to assess risk of bias in the included studies, including details of the tool(s) used, how many reviewers assessed each study and whether they worked independently, and if applicable, details of automation tools used in the process. | Page 5/6 |
| Effect measures | 12 | Specify for each outcome the effect measure(s) (e.g. risk ratio, mean difference) used in the synthesis or presentation of results. | Page 5/6 |
| Synthesis methods | 13a | Describe the processes used to decide which studies were eligible for each synthesis (e.g. tabulating the study intervention characteristics and comparing against the planned groups for each synthesis (item #5)). | Page 7/8 |
|  | 13b | Describe any methods required to prepare the data for presentation or synthesis, such as handling of missing summary statistics, or data conversions. | Page 7/8 |
|  | 13c | Describe any methods used to tabulate or visually display results of individual studies and syntheses. | Page 7/8 |
|  | 13d | Describe any methods used to synthesize results and provide a rationale for the choice(s). If meta-analysis was performed, describe the model(s), method(s) to identify the presence and extent of statistical heterogeneity, and software package(s) used. | Page 7/8 |
|  | 13e | Describe any methods used to explore possible causes of heterogeneity among study results (e.g. subgroup analysis, meta-regression). | Page7/8 |
|  | 13f | Describe any sensitivity analyses conducted to assess robustness of the synthesized results. | N/A |
| Reporting bias assessment | 14 | Describe any methods used to assess risk of bias due to missing results in a synthesis (arising from reporting biases). | Page 7/8 |
| Certainty assessment | 15 | Describe any methods used to assess certainty (or confidence) in the body of evidence for an outcome. | Page 7/8 |
| **RESULTS** | | |  |
| Study selection | 16a | Describe the results of the search and selection process, from the number of records identified in the search to the number of studies included in the review, ideally using a flow diagram. | Page 5/6 |
|  | 16b | Cite studies that might appear to meet the inclusion criteria, but which were excluded, and explain why they were excluded. | Page 5/6 |
| Study characteristics | 17 | Cite each included study and present its characteristics. | Page 8-14 |
| Risk of bias in studies | 18 | Present assessments of risk of bias for each included study. | Page 8-14 |
| Results of individual studies | 19 | For all outcomes, present, for each study: (a) summary statistics for each group (where appropriate) and (b) an effect estimate and its precision (e.g. confidence/credible interval), ideally using structured tables or plots. | Page 8-14 |
| Results of syntheses | 20a | For each synthesis, briefly summarise the characteristics and risk of bias among contributing studies. | Page 8-14 |
|  | 20b | Present results of all statistical syntheses conducted. If meta-analysis was done, present for each the summary estimate and its precision (e.g. confidence/credible interval) and measures of statistical heterogeneity. If comparing groups, describe the direction of the effect. | Page 8-14 |
|  | 20c | Present results of all investigations of possible causes of heterogeneity among study results. | Page 8-14 |
|  | 20d | Present results of all sensitivity analyses conducted to assess the robustness of the synthesized results. | N/A |
| Reporting biases | 21 | Present assessments of risk of bias due to missing results (arising from reporting biases) for each synthesis assessed. | Page 14 |
| Certainty of evidence | 22 | Present assessments of certainty (or confidence) in the body of evidence for each outcome assessed. | Page 14 |
| **DISCUSSION** | | |  |
| Discussion | 23a | Provide a general interpretation of the results in the context of other evidence. | Page 14-17 |
|  | 23b | Discuss any limitations of the evidence included in the review. | Page 14-17 |
|  | 23c | Discuss any limitations of the review processes used. | Page 14-17 |
|  | 23d | Discuss implications of the results for practice, policy, and future research. | Page 14-17 |
| **OTHER INFORMATION** | | |  |
| Registration and protocol | 24a | Provide registration information for the review, including register name and registration number, or state that the review was not registered. | Page 3 |
|  | 24b | Indicate where the review protocol can be accessed, or state that a protocol was not prepared. | Page 3 |
|  | 24c | Describe and explain any amendments to information provided at registration or in the protocol. | Page 3 |
| Support | 25 | Describe sources of financial or non-financial support for the review, and the role of the funders or sponsors in the review. | Page 60 |
| Competing interests | 26 | Declare any competing interests of review authors. | Page 60 |
| Availability of data, code and other materials | 27 | Report which of the following are publicly available and where they can be found: template data collection forms; data extracted from included studies; data used for all analyses; analytic code; any other materials used in the review. | All additional data can be found in the supplemental documentation |

*From:*  Page MJ, McKenzie JE, Bossuyt PM, Boutron I, Hoffmann TC, Mulrow CD, et al. The PRISMA 2020 statement: an updated guideline for reporting systematic reviews. BMJ 2021;372:n71. doi: 10.1136/bmj.n71

For more information, visit: <http://www.prisma-statement.org/>

**Supplemental Table 3: reported frequency and risk of workplace violence encountered by HCWs in ICUs**

| **Author** | **Level of evidence** | **Physical violence** | **Verbal violence** | **Sexual harassment** | **Main Perpetrators** | **Violence compared in ICUs to other wards** |
| --- | --- | --- | --- | --- | --- | --- |
| Barrass 2019 [35] | 4 | 57% | 63% | Not reported | Patients, visitors | Not reported (Only included ICU staff) |
| De Araujo 2018 [44] | 4 | 25% | 73% | 12% | Mostly patient and family except for coworkers/boss for sexual abuse | Not reported (Only included ICU staff) |
| Ferns 2002 [46] | 4 | 68% | Not reported | Not reported | Patient, visitors | Not reported (Only included ICU staff) |
| Kumar 2019 [61] | 4 | 10% | 36% | Not reported | Visitors | Not reported (Only included ICU staff) |
| Lynch 2003 [63] | 4 | 77% | 87% | Not reported | Patients, relatives | Not reported (Only included ICU staff) |
| Parke 2022 [69] | 4 | Not reported | 57% | 13% | Patients, other nurses | Not reported (Only included ICU staff) |
| Pol 2019 [17] | 3c | Not reported | Not reported | Not reported | Patients | Not reported (Only included ICU staff) |
| Schiltz 2020 [71] | 4 | 16% | 17% | Not reported | Patients | Not reported (Only included ICU staff) |
| Towhari 2020 [73] | 4 | 31% | 46% | Not reported | Patients | Not reported (Only included ICU staff) |
| Ulrich 2022 [74] | 4 | 28% | 65% | Not reported | Patients, families | Not reported (Only included ICU staff) |
| Wang 2022 [82] | 4 | 78% | 52% | Not reported | Patients | Not reported (Only included ICU staff) |
| Yi 2022 [78] | 4 | 10% | 19% | 7% | Patients | Not reported (Only included ICU staff) |
| Yoo 2018 [79] | 4 | 60% | 90% | Not reported | Patients, relatives | Not reported (Only included ICU staff) |
| Rodriguez-Acosta 2010 [25] | 3b | Not reported | Not reported | Not reported | Patients | Relative risk of experiencing violence was higher in the ICU (RR 2.3 (95% CI=1-4) when compared to obstetrics/pediatrics. In general, lower RR than staff in psychiatry but higher RR than ED staff. |
| Abdul Rahman 2017 [30] | 4 | 24% | 25% | 21% | Patients and relatives | Odds for experiencing threats of violence 4 times higher in ER nurses vs. ICU nurses (95% CI= 2.2-7.3) |
| Ahmed 2012 [31] | 4 | 18%^1^ | 37%^1^ | Not reported | Patients and relatives, then nurses/doctors | Higher frequencyy of abuse in ICU and ED compared to general wards |
| Al-Nemari 2020 [32] | 4 | 52%^2^ | 52%^2^ | Not reported | Patients | Odds to experience abuse in ED 3.5 times higher than in ICU (95% CI= 1.95-6.2) |
| Alfuqaha 2022 [33] | 4 | 5% for physicians, 19% for nurses | 10% for physicians, 19% for nurses | Not reported | Patients | Violence significantly more common in ED compared to ICU |
| Alquwez 2020 [34] | 4 | Not reported | Not reported | Not reported | Patients, other nurses | Nurses in ED and outpatient departments had higher incivility scores than nurses in ICUs |
| Alshehry 2019 [96] | 4 | Not reported | Not reported | Not reported | Patients, physicians and other nurses | Nurses in Medical-Surgical departments reported significantly more experience of incivility than ICU nurses |
| Alshehry 2022 [92] | 4 | Not reported | Not reported | Not reported | Patient and family | Nurses in Medical-Surgical departments and EDs perceived greater conflict than those in ICUs |
| Boafo 2017 [36] | 4 | 9%^1^ | Not reported | Not reported | Most often relatives | No statistical association depending on workplace |
| Byon 2021 [37] | 4 | 44% | 68% | Not reported | Not reported | Lower OR (0.86 (95% CI=0.4-18.6)) for ICU nurses for experiencing violence and verbal threats when compared to ED nurses |
| Camerino 2008 [38] | 4 | 21% | Not reported | Not reported | Patients | Lower frequency of violence in the ICU when compared to the ED (no statistical analysis) |
| Chang 2019 [91] | 4 | Not reported | 74^1^ | Not reported | Not reported | Level of aggression did not differ by unit type, trend for higher rates in ICUs and EDs |
| Chen 2018 [39] | 4 | 2% | 37% | Not reported | Relatives and visitors | Higher odds of experiencing non-physical violence in the ICU compared to paramedical staff (OR 2 (95% CI=1.6-2.6)) |
| Chen 2022 [40] | 4 | 16%^1^ | 49%^1^ | 16% verbal, 8% assault^1^ | Not reported | Higher likelihood of burnout in ICU nurses after experiencing violence compared to mental health nurses (OR 4.8 (95% CI=1.9-12)) |
| Cheraghi 2012 [41] | 4 | Up to 14% | Up to 72% | 0% | Patients, relatives | Higher frequency of physical violence in ICUs compared to ED and the dialysis unit |
| Cho 2020 [42] | 4 | 78%^1,2^ | Not reported | Not reported | Patients, relatives | Significant less violence exposure in the ICU compared to step down units and general wards |
| Chowdhury 2022 [43] | 4 | 26%^1^ | 72%^1^ | 6%^1^ | Not reported | ED nurses more frequently experience physical violence compared to ICU nurses |
| Duncan 2001 [45] | 4 | 21%^1^ | 38%^1^ | 8%^1^ | Patients, visitors | ED or psychiatry were factors in multiple regression modelling for higher risk of experiencing violence but not ICU |
| Findorff 2004 [47] | 4 | 7%^1^ | 31%^1^ | Not reported | Patients, coworkers | Increased odds of experiencing physical violence in the ICU compared to medical wards (OR 4.9 (95% CI=2.6-9.2)) |
| Fujita 2012 [48] | 4 | 29% | 35% | 13% | Not reported | Odds of being a victim of physical aggression were significantly higher in the ICU compared to general wards (OR 2.44 (95% CI=1.6-3.7)) |
| Gerberich 2004 [49] | 4 | 13%^1^ | 38%^1^ | Not reported | Patients | Higher odds of experiencing physical and non-physical violence in the ICU compared to Medical-Surgical and Obstetric wards (OR 1.57 (95% CI=1.1-2.2)) |
| Grover 2020 [50] | 4 | 53%^2^ | Not reported | Not reported | Relatives, Patients, Staff | No statistical significance in violence rates between different departments reported |
| Guimaraes 2019 [51] | 4 | Not reported | Not reported | Not reported | Patients | Offensive behavior was more common in the ED than in the ICU |
| Guo 2022 [52] | 4 | 4% | 22% | Not reported | Patients, visitors | Staff in the ICU had a higher likelihood of experiencing violence when compared to staff in the general wards (OR 22.9 (95% CI=2.9-181)) |
| Hahn 2013 [53] | 4 | 17%^1^ | 46%^1^ | Not reported | Patients, visitors | Higher odds to experience violence in the ICU when compared to medical wards (OR 1.4 (95% CI=1.1-1.8)) |
| Hamzaoglu 2019 [54] | 4 | 29% | 77% | Not reported | Patients, Visitors | Higher frequency of violence in the ED when compared to the ICU |
| Harati 2022 [55] | 4 | Not reported | Not reported | Not reported | Patients, visitors | No statistical difference in aggression between different units |
| Havaei 2020 [56] | 4 | 60% | 79% | 42% harassment, 5% assault | Patients, families, visitors, other nurses | Nurses in ICU were less frequently exposed to all types of violence when compared to ED/Psychiatry/general wards |
| Hesketh 2003 [57] | 4 | 12% | 28% | 5% harassment, 0.7% assault | Patients, coworkers | Lower frequency of violence in the ICU when compared to other units. ICU nurses were more likely to experience emotional abuse and sexual harassment from a colleague than other specialties |
| Ihara 2010 [58] | 4 | 80% | 85% | Not reported | Patients | Nurses in the ED and ICU experienced higher frequency of violence |
| Jiao 2015 [59] | 4 | 17% | 75% | Not reported | Patients, relatives | No statistically significant lower odds of experiencing violence in the ICU when compared to the ED |
| Kim 2022 [60] | 4 | 32%^1^ | 56%^1^ | Not reported | Patients, visitors | Higher odds for ICU staff to experience violence when compared to medical wards (OR 1.57 (95% CI= 1.1-2.2)) |
| Layne 2019 [62] | 4 | Not reported | Not reported | Not reported | Patients, families | ICU nurses reported significantly more incivility experienced than other specialties |
| Lepiesova 2015 [94] | 4 | 83% | 97% | 68% | Patients | ICU, ED and psychiatry nurses experience significantly more violence than nurses in other wards |
| Munoz 2021 [64] | 4 | 0% | 85% | 0% | Patients, violence | Working in a COVID-19 ICU was associated with a higher likelihood of experiencing violence (OR 5.8 (95% CI=2-21)) |
| Musengamana 2022 [65] | 4 | 7% | 55% | 2% | Patients | No significant association with experiencing more violence in the ICU when compared to the ED and HIV Unit |
| Ogenler 2018 [66] | 4 | 29% | Not reported | Not reported | Patients, relatives | ED nurses experienced higher frequency of violence when compared to ICU nurses |
| Pandey 2018 [67] | 4 | 16%^1^ | 62%^1^ | 9%^1^ | Relatives, Patients | No statistically significant difference between violence experience in the ICU and other units |
| Park 2015 [68] | 4 | 49% | 76% | 23% | Patients, families | Physical violence, threats of violence and verbal abuse occurred most frequently in ICUs |
| Perkins 2020 [70] | 4 | 69% | 90% | Not reported | Patients, relatives | Critical care nurses had an increased perception of physical assault when compared with perinatal services. |
| Shafran-Tikva 2017 [93] | 4 | 76%^2^ | Not reported | Not reported | Patients, relatives | Nurses in the ICU were less likely to experience violence when compared to ED nurses (OR 0.22 (95% CI=0.04-1.2)). Physicians experience of violence did not differ between units |
| Sharma 2019 [72] | 4 | 4% | 50% | Not reported | Not reported | More incidents recorded in the ICU compared to the ED |
| Ünsal 2013 [75] | 4 | 11% | 38% | Not reported | Patients, visitors | Higher frequency of verbal violence in the ICU when compared to the ED but similar frequency of physical violence. Less violence than in psychiatric units. |
| Wang 2021 [76] | 4 | 8% | 38% | Not reported | Patients, gangs | ICU workers were more likely to experience violence when compared to general ward nurses (OR 2.4 (95% CI=1.3-4.1)). Similar odds when compared to ED nurses. |
| Wei 2015 [77] | 4 | 31% | 49% | Not reported | Patients | Nurses in the ICU were more likely to experience violence when compared to nurses in the outpatient departments. (OR 6.25 (95% CI=5.4-7.2)) |
| Zhang 2017 [80] | 4 | 23% | 53% | 3% | Patients | ICU workers had higher odds of experiencing physical violence when compared to obstetric nurses (OR 1.9 (95% CI=1.2-3)) |

**^1^**No specific numbers for ICU staff alone ^2^No differentiation between types of violence

**Supplemental Table 4: Staff demographics and patient characteristics associated with workplace violence**

| **Author** | **Level of evidence** | **Staff demographics associated with experiencing workplace violence** | **Characteristics associated with workplace violence** |
| --- | --- | --- | --- |
| Nikstaitis 2014 [24] | 3b | White people perceived less violence when compared to black people. Younger nurses and nurses that have been practicing for 5 or more years were independent predictors of experiencing incivility. | Not reported |
| Rodriguez-Acosta 2010 [25] | 3b | Younger workers and workers with shorter experience had higher risk of being assaulted. Black workers had a lower risk of being assaulted when compared to non-black workers. | Not reported |
| Pol 2019 [17] | 3c | Male nursing staff were found to be more likely involved in incidents of verbal violence. Female nurses were most likely to experience overall violence. | Patients having taken a drug overdose or received a head trauma were more likely to cause violence. |
| Slack 2022 [26] | 3c | Not reported | Violent patients were more likely to have been staying in the hospital or the ICU more than one week. Age, APACHE II score and mortality did not differ between violent and non-violent patients. |
| Tachibana 2021 [27] | 3c | Not reported | Older age, male gender and active smoking status were identified as possible risk factors associated with violence in patients with delirium |
| Thomas 2015 [28] | 3c | Nurses were the most common victims of the reported incidents. | 4% of incidents related to illness and confusion. |
| Ahmed 2012 [31] | 4 | Younger and female nurses were more likely to experience abuse | Not reported |
| Al-Nemari 2020 [32] | 4 | Younger staff had higher rates of abuse. There was no difference in gender, shift work or professional role and rates of violence. | Not reported |
| Alfuqaha 2022 [33] | 4 | Nurses more often physically abused, while physicians are more often verbally abused. Younger and female staff more likely to experience abuse according to logistic regression. | Not reported |
| Alshehry 2019 [96] | 4 | Foreign nationality correlated with experiencing more incivility from patients | Not reported |
| Alshehry 2022 [92] | 4 | Not reported | Family perceived that staff used insensitive or offensive language. The family had distrust of the motivations of the staff members. Contradictory communication. |
| Boafo 2017 [36] | 4 | Staff in regional hospitals more likely to experience violence when compared to bigger hospitals. No difference in age. | Not reported |
| Byon 2021 [37] | 4 | Nurses who cared for COVID-19 patients were more likely to experience violence than nurses who did not look after these patients. | Understaffing, higher stress during COVID-19 pandemic |
| Camerino 2008 [38] | 4 | Higher rates of violence reported by younger and male nurses. Foreign staff and shift workers experienced higher rates of violence. | Not reported |
| Chen 2018 [39] | 4 | Younger and female nurses were more likely to experience any type of violence. | Nurse-patient miscommunication. Negative image of healthcare staff due to media. Patients blaming nurses for conflicts with physicians. |
| Cho 2020 [42] | 4 | Male nurses reported higher rates of abuse | Not reported |
| Chowdhury 2022 [43] | 4 | Male nurses, nurses working extended hours and non-trained nurses were more likely to experience physical violence. Nurses working in public hospitals were more likely to experience verbal violence. | Not reported |
| Duncan 2001 [45] | 4 | Younger age correlated with more violence exposure. There was less violence if there was prevention measures in place. | Not reported |
| Findorff 2004 [47] | 4 | Nurses and staff with high patient contact were more likely to experience violence compared to physicians and staff with less patient contact. Increased supervisor support decreased the likelihood of experiencing violence. | 68% of physically violent patients were perceived to be impaired by disease, medications, durgs or alcohol. For verbally abusive patients only 10% were perceived to be impaired. |
| Gerberich 2004 [49] | 4 | Not reported | 80% of physically violent patients were impaired by their illness or medication. For verbal violence only 41% of the patients seemed impaired. Typically violent patients were male and 66 years or older. |
| Grover 2020 [50] | 4 | Younger age and less experience were risk factors for experiencing workplace violence. | Long waiting periods. Unrealistic expectations of patients and relatives. Poor communication skills of doctors. |
| Guo 2022 [52] | 4 | Nurses had higher OR to experience verbal violence than doctors and other staff. Doctors were more likely to experience physical violence. | Not reported |
| Hahn 2013 [53] | 4 | Higher odds of experiencing violence when staff is younger and does not believe in preventive measures. | Patients over the age of 65 years are more likely to be violent, often accompanied by delirium or dementia |
| Hamzaoglu 2019 [54] | 4 | Doctors more often experience violence when compared to nurses. Female staff are more often victims of physical violence | Not reported |
| Havaei 2020 [56] | 4 | Nurses with more patient contacted reported higher rates of experiencing violence | Not reported |
| Hwang 2015 [81] | 4 | Younger nurses felt like they experienced more violence | Not reported |
| Jiao 2015 [59] | 4 | More experienced nurses and nurses with lower anxiety levels were less likely to experience violence | Organized crime in China |
| Kim 2022 [60] | 4 | Higher rates of experiencing physical violence in nurses when compared to physicians. Visitors more often verbally abused nurses than other staff. | Not reported |
| Kumar 2019 [61] | 4 | Higher rates of violence during the night when less staff was available. | Poor communication, billing disputes, dissatisfaction with medical services were major causes for violent behaviour. |
| Lepiesova 2015 [94] | 4 | Nurses with shift work experienced more violence when compared to nurses working normal shifts. | Not reported |
| Lynch 2003 [63] | 4 | Not reported | Relatives in distress, alcohol consumption and sociopathic behaviour were the main causes of visitor violence. For patients 72% of violent episodes were attributed to illness or the treatment. |
| Munoz 2021 [64] | 4 | Higher odds of experiencing violence in female physicians compared to their male counterparts. | Shortcomings of the healthcare services were often triggers for violence. |
| Musengamana 2022 [65] | 4 | No association of violence with age, gender and number of staff. | No association of patient age and sex of patient with violence. |
| Ogenler 2018 [66] | 4 | No significant relation of experience of violence with age, gender, work experience or working hours. | Not reported |
| Patterson 2022 [16] | 4 | Not reported | Patients with comorbid mental illness |
| Pandey 2018 [67] | 4 | Nurses working in shifts, especially night shifts, and divorced or separated nurses were more likely to experience violence | Not reported |
| Park 2015 [68] | 4 | Nurses with high work demands were more often victims of violence | Not reported |
| Parke 2022 [69] | 4 | Younger age and ethnicity associated with experiencing sexual harassment | Not reported |
| Perkins 2020 [70] | 4 | Not reported | 24% of violent patients or relatives showed altered neurocognitive function (dementia, delirium, drugs) |
| Schiltz 2020 [71] | 4 | Smaller sized ICUs had lower odds of violence than larger ICUs. Nurses were four times more likely to be verbally threatened and seven times more likely to be physically assaulted compared to physicians. | Not reported |
| Shafran-Tikva 2017 [93] | 4 | Nurses were exposed more often to violence than physicians. The higher the rank of the physician the lower the experience of violence. Younger nurses and physicians were more likely to be victims of violence | Not reported |
| Sharma 2019 [72] | 4 | Higher number of violent events in the younger age groups. | Unexpected death or complications, extended hospital stay, staff shortages and billing disputes were perceived to be responsible for the events of violence. |
| Wang 2021 [76] | 4 | Staff between 41 and 45 years old, physicians and staff in rural hospitals were more likely to experience violence when compared to their counterparts. | Not reported |
| Wang 2022 [82] | 4 | Male nurses, nurses with less job experience and lower ranked nurses had higher odds of experiencing violence. | Failure to meet unreasonable requirements was a risk factor for violence |
| Wei 2015 [77] | 4 | Male nurses had higher rates of experience of physical violence. More educated nurses were more likely to experience verbal violence but not physical violence. Younger nurses were more likely to experience violence compared to older nurses. | Not reported |
| Yi 2022 [78] | 4 | Longer weekly hours and lower work experience put staff at higher odds of experiencing violence. | Miscommunication, long waiting times and unsatisfactory treatment effect. |
| Zhang 2017 [80] | 4 | Male staff, less experienced workers and rotating shifts were associated with a higher prevalence of physical violence. | Not reported |

**Supplemental Table 5: Frequency of underreporting violent incidents as discovered by interviews with healthcare staff and reasons for underreporting**

| **Author** | **Level of evidence** | **Frequency of failure to report incidents** | **Reasons for not reporting** |
| --- | --- | --- | --- |
| Ahmed 2012 [31] | 4 | 65% | Not reported |
| Al-Nemari 2020 [32] | 4 | 33% | No reporting system in place |
| Alfuqaha 2022 [33] | 4 | 60% | Not reported |
| Boafo 2017 [36] | 4 | 64% | Not reported |
| Byon 2021 [37] | 4 | Not reported | 10% reported difficulties reporting incidents due to COVID-19 pandemic |
| Cheraghi 2012 [41] | 4 | 20% | Not reported |
| De Araujo 2018 [44] | 4 | 85% | Fear of being fired, shame, fear of judgement, fear of the aggressor. |
| Duncan 2001 [45] | 4 | 70% | Not reported |
| Gerberich 2004 [49] | 4 | 30% | Non-supportive environment, too busy with work, regarded it as a minor or isolated event and regarded it as part of the job. |
| Grover 2020 [50] | 4 | 23% | Lack of time, difficult to report, uselessness of reporting incidents. |
| Hamzaoglu 2019 [54] | 4 | Most incidents reported | Up to 62% reported nothing happened after they reported an incident |
| Kumar 2019 [61] | 4 | 15% | Non-satisfactory response in 53% |
| Lynch 2003 [63] | 4 | 44% | Only events leading to significant injury were recorded in some ICUs, some ICUs did not possess recording systems or forms |
| Ogenler 2018 [66] | 4 | 18% | Not reported |
| Parke 2022 [69] | 4 | 75% | Not reported |
| Perkins 2020 [70] | 4 | 45% | Not reported |
| Towhari 2020 [73] | 4 | 46% | Regarded as part of job, privacy concerns, no knowledge of reporting system and no training for reporting systems |
| Wang 2022 [82] | 4 | 57% | Regarded as part of the job, nothing will change after reporting |
| Yi 2022 [78] | 4 | 65% | Not reported |
| Yoo 2018 [79] | 4 | 34% | Not reported |

**Supplemental Table 6: Assessment of included cohort studies according to the Newcastle-Ottawa Quality Assessment Form**

| **Author** | **Selection** | **Comparability** | **Outcome** | **Quality** |
| --- | --- | --- | --- | --- |
| Nikstaitis 2014 [24] | ** | * | ** | Poor Quality |
| Pol 2019 [17] | **** | ** | ** | Good Quality |
| Rodriguez-Acosta 2010 [25] | *** | ** | ** | Good Quality |
| Slack 2022 [26] | *** | ** | ** | Good Quality |
| Sona 2022 [29] | ** | * | * | Poor quality |
| Tachibana 2021 [27] | ** | ** | ** | Fair Quality |
| Thomas 2015 [28] | ** | * | ** | Fair Quality |

**Supplemental Table 7: Assessment of included cross-sectional studies according to the MIXED METHODS APPRAISAL TOOL (MMAT)**

| **Author** | **Representative sample^1^** | **Measurements appropriate^2^** | **Risk of nonresponse bias^3^** | **Other bias** |
| --- | --- | --- | --- | --- |
| Abdul Rahman 2017 [30] | Somewhat | Appropriate | Yes | Recall bias, reporting bias |
| Ahmed 2012 [31] | Somewhat | Appropriate | No | Recall bias, selection bias |
| Al-Nemari 2020 [32] | Yes | Appropriate | No | Recall bias |
| Alfuqaha 2022 [33] | Somewhat | Appropriate | Yes | Recall bias, reporting bias, selection bias |
| Alquwez 2020 [34] | Somewhat | Appropriate | Yes | Recall bias, reporting bias |
| Alquwez 2023 [84] | Somewhat | Appropriate | Yes | Recall bias, reporting bias |
| Alshehry 2019 [96] | Somewhat | Appropriate | Yes | Recall bias, reporting bias, selection bias |
| Alshehry 2022 [92] | Somewhat | Appropriate | Yes | Recall bias, reporting bias |
| Barrass 2019 [35] | Somewhat | No | Yes | Abstract, recall bias, selection bias, reporting bias |
| Boafo 2017 [36] | No | Appropriate | Yes | Recall bias, reporting bias, selection bias |
| Byon 2021 [37] | Somewhat | Appropriate | Yes | Selection bias, recall bias, reporting bias |
| Camerino 2008 [38] | Yes | Appropriate | Yes | Recall bias, reporting bias, loss-to-follow up |
| Cha 2020 [95] | Somewhat | Appropriate | No | Recall bias, selection bias |
| Chang 2019 [91] | Somewhat | Appropriate | No | Selection bias, recall bias |
| Chen 2018 [39] | Yes | Appropriate | No | Recall bias, selection bias |
| Chen 2022 [40] | Yes | Appropriate | No | Recall bias, selection bias |
| Cheraghi 2012 [41] | No | Appropriate | Yes | Reporting bias, recall bias, selection bias |
| Cho 2020 [42] | Somewhat | Appropriate | No | Recall bias, selection bias |
| Chowdhury 2022 [43] | Somewhat | No | No | Selection bias, recall bias |
| De Araujo 2018 [44] | Somewhat | No | Yes | Recall bias, selection bias, reporting bias |
| Duncan 2001 [45] | Yes | No | Yes | Recall bias, reporting bias |
| Ferns 2002 [46] | No | No | No | Selection bias, recall bias |
| Findorff 2004 [47] | Yes | No | Yes | Recall bias, selection bias, reporting bias |
| Fujita 2012 [48] | Yes | No | Yes | Recall bias, selection bias |
| Garzon 2022 [83] | No | No | Yes | Recall bias, selection bias, reporting bias |
| Gerberich 2004 [49] | Yes | No | Yes | Recall bias, reporting bias |
| Grover 2020 [50] | Somewhat | Appropriate | No | Recall bias, selection bias |
| Guimaraes 2019 [51] | No | Appropriate | Yes | Recall bias, selection bias, reporting bias |
| Guo 2022 [52] | Yes | Appropriate | No | Recall bias, reporting bias |
| Hahn 2013 [53] | Somewhat | Appropriate | Yes | Recall bias, reporting bias |
| Hamzaoglu 2019 [54] | Somewhat | Appropriate | No | Recall bias, selection bias |
| Harati 2022 [55] | Somewhat | Appropriate | No | Recall bias, selection bias |
| Havaei 2020 [56] | Yes | No | Yes | Recall bias, reporting bias, selection bias |
| Hesketh 2003 [57] | Yes | No | Yes | Recall bias, selection bias |
| Hwang 2015 [81] | No | No | No | Recall bias, selection bias, reporting bias |
| Ihara 2010 [58] | Somewhat | No | No | Recall bias, reporting bias |
| Jeong 2018 [85] | Somewhat | Appropriate | Yes | Recall bias, selection bis, reporting bias |
| Jeong 2021 [86] | Somewhat | Appropriate | Yes | Recall bias, selection bias, reporting bias |
| Jiao 2015 [59] | Somewhat | Appropriate | Yes | Recall bias, selection bias, reporting bias |
| Keys 2008 [88] | No | No | Yes | Recall bias, selection bias, reporting bias |
| Kim 2022 [60] | Somewhat | Appropriate | Yes | Recall bias, reporting bias |
| Kumar 2019 [61] | Somewhat | No | Yes | Recall bias, selection bias, reporting bias |
| Layne 2019 [62] | Somewhat | Appropriate | Yes | Recall bias, reporting bias, selection bias |
| Lepiesova 2015 [94] | Yes | Appropriate | No | Recall bias, reporting bias |
| Lykins 2021 [87] | Somewhat | No | Yes | Recall bias, reporting bias, selection bias |
| Lynch 2003 [63] | Somewhat | No | Yes | Recall bias, reporting bias, selection bias |
| Mason 2014 [90] | No | No | Yes | Recall bias, reporting bias, selection bias |
| Munoz 2021 [64] | Somewhat | Appropriate | No | Recall bias, reporting bias |
| Musengamana 2022 [65] | Somewhat | Appropriate | No | Recall bias, selection bias, reporting bias |
| Ogenler 2018 [66] | Somewhat | No | Yes | Recall bias, selection bias, reporting bias |
| Pandey 2018 [67] | Somewhat | Appropriate | No | Recall bias, selection bias, reporting bias |
| Park 2015 [68] | Somewhat | Appropriate | Yes | Recall bias, selection bias, reporting bias |
| Parke 2022 [69] | Yes | No | No | Recall bias, selection bias |
| Patterson 2022 [16] | No | No | Yes | Recall bias, selection bias, reporting bias |
| Perkins 2020 [70] | Somewhat | Appropriate | Yes | Recall bias, selection bias, reporting bias |
| Schiltz 2020 [71] | Yes | No | Yes | Recall bias, reporting bias |
| Shafran-Tikva 2017 [93] | Somewhat | No | Yes | Recall bias, selection bias, reporting bias |
| Sharma 2019 [72] | Somewhat | Appropriate | No | Recall bias, selection bias, reporting bias |
| Towhari 2020 [73] | Somewhat | No | No | Recall bias, reporting bias |
| Ulrich 2022 [74] | Yes | Appropriate | Yes | Recall bias, reporting bias, |
| Ünsal 2013 [75] | Somewhat | No | Yes | Recall bias, selection bias, reporting bias |
| Wang 2021 [76] | Yes | Appropriate | Yes | Recall bias, selection bias, reporting bias |
| Wang 2022 [82] | Somewhat | Appropriate | Yes | Recall bias, selectino bias, reporting bias |
| Wei 2015 [77] | Yes | Appropriate | Yes | Recall bias, reporting bias |
| Wood 2019 [89] | No | No | Yes | Reporting bias, selection bias |
| Yi 2022 [78] | Somewhat | No | Yes | Recall bias, selection bias, reporting bias |
| Yoo 2018 [79] | Somewhat | Appropriate | Yes | Recall bias, selection bias |
| Zhang 2017 [80] | Yes | Appropriate | Yes | Recall bias, selection bias, reporting bias |

**^1^ Deemed representative when it was multicentric and had a big sample size, deemed somewhat representative if sample size was appropriate ^2^ Measurement was deemed appropriate if used previously validated questionnaire ^3^ Cut-off for nonresponse bias was a response rate under 80%**

**Supplemental Figure 1: Meta-analysis of frequency of physical violence**

**
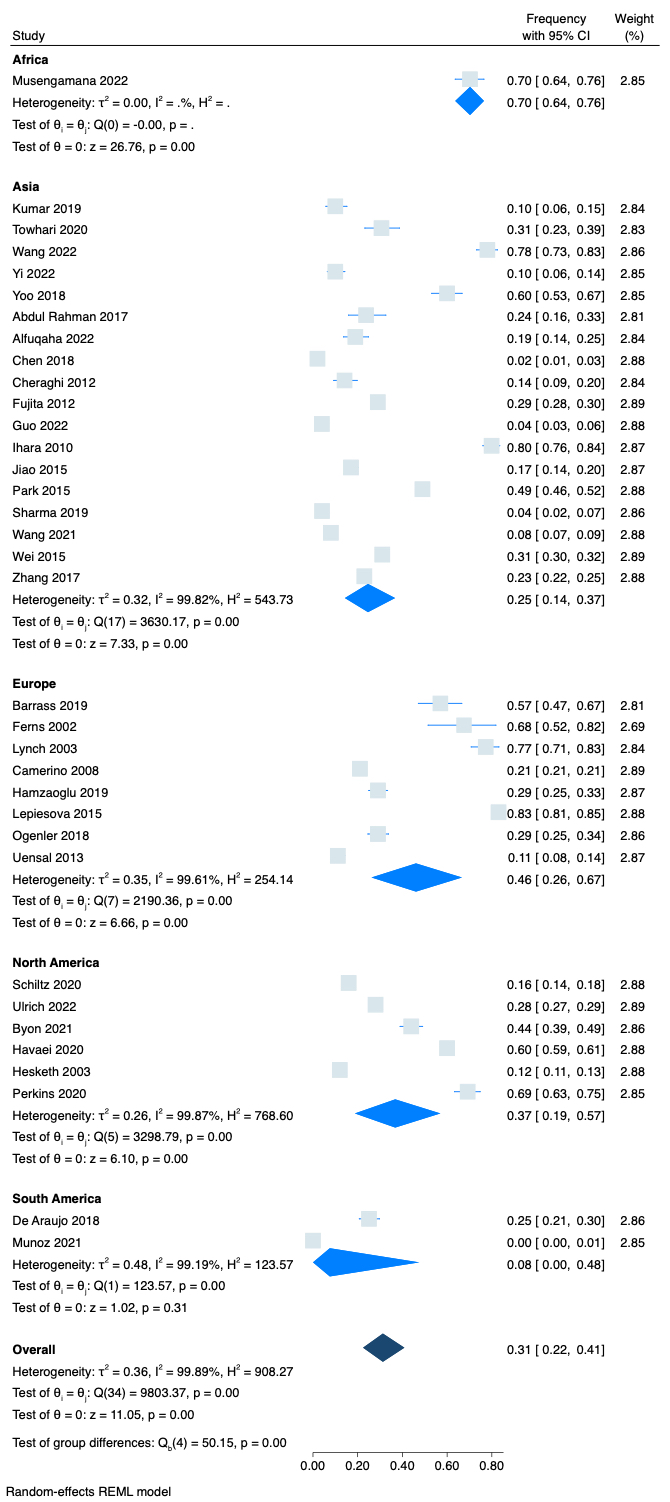
**

**Supplemental Figure 2: Meta-analysis of frequency of verbal violence**

**
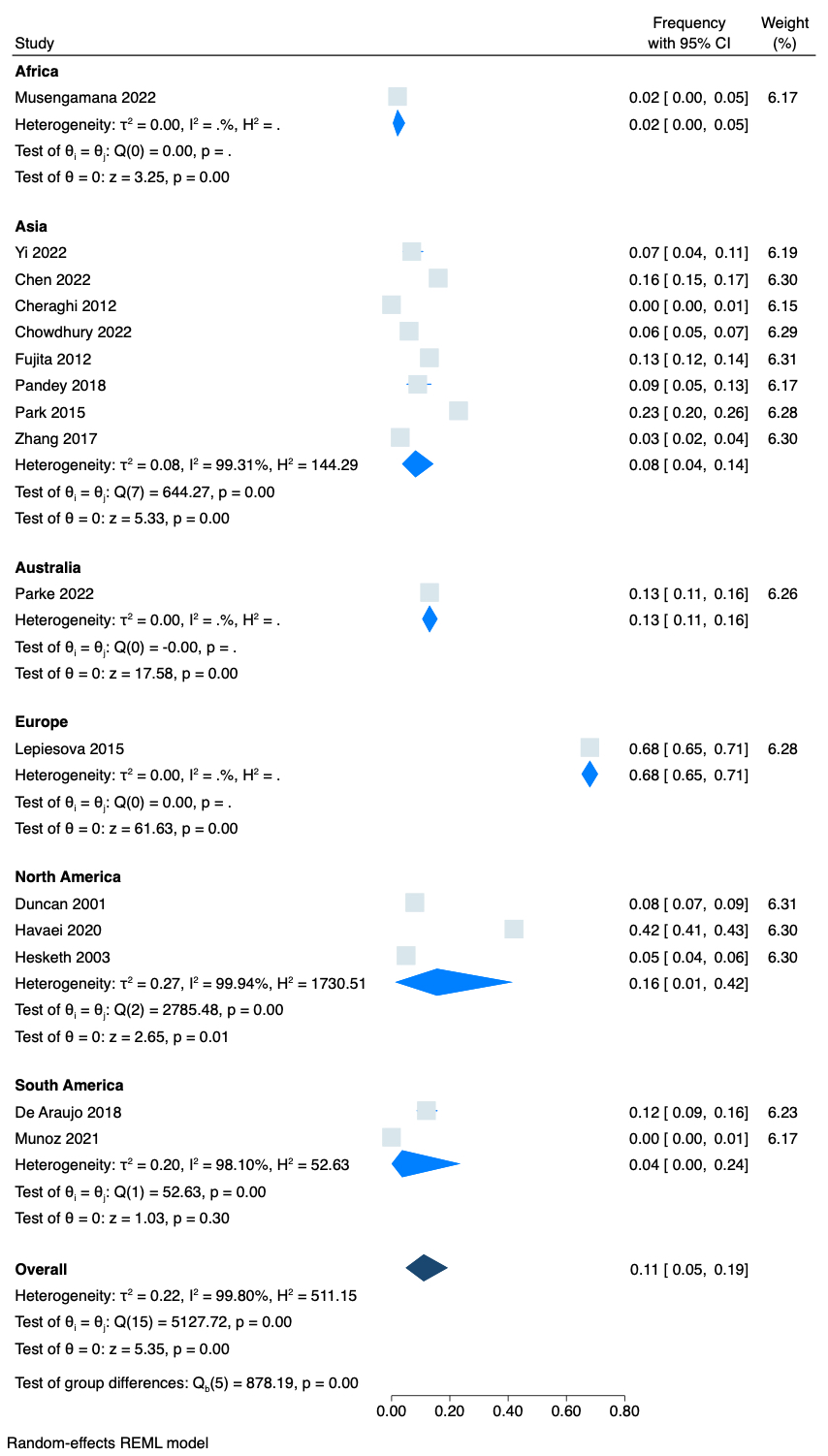
**

**Supplemental Figure 3: Meta-analysis of frequency of sexual violence**

**
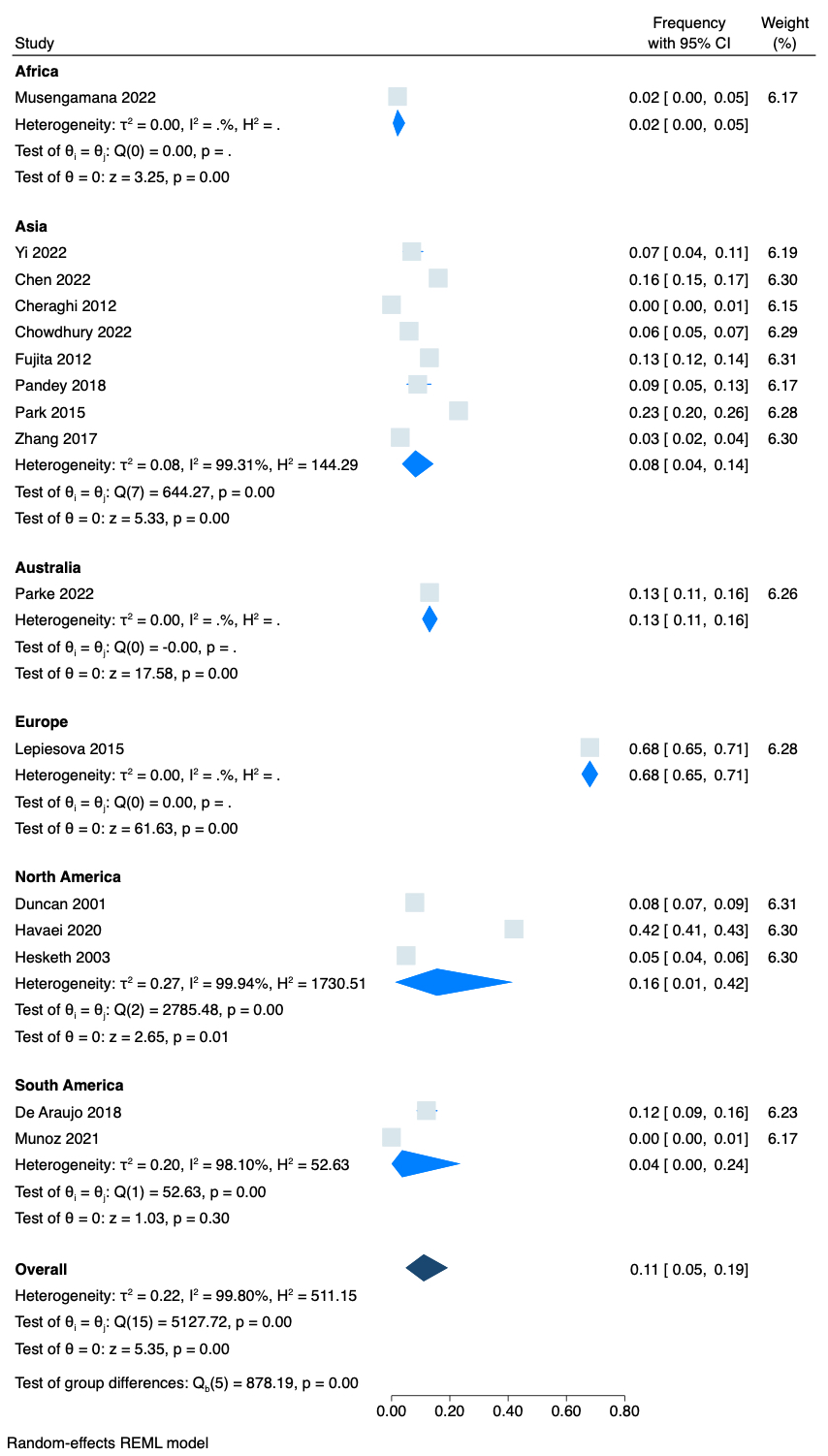
**

**Supplemental Figure 4: Meta-analysis of frequency of underreporting of violent events**

**
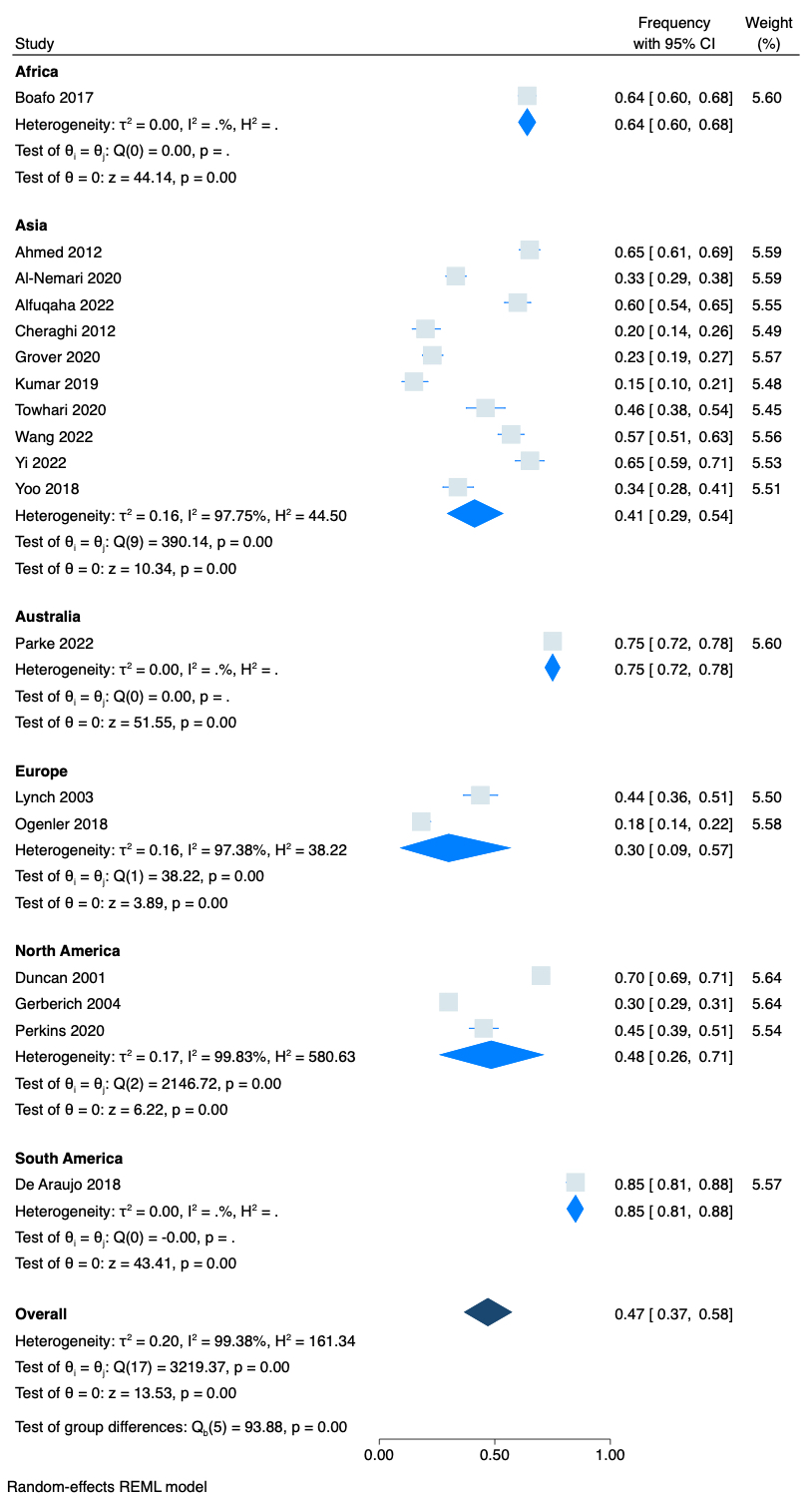
**

**Supplemental Figure 5: LFK index for asymmetry for meta-analysis of (A) physical violence (B) verbal violence (C) sexual violence and (D) underreporting of violent events**

**
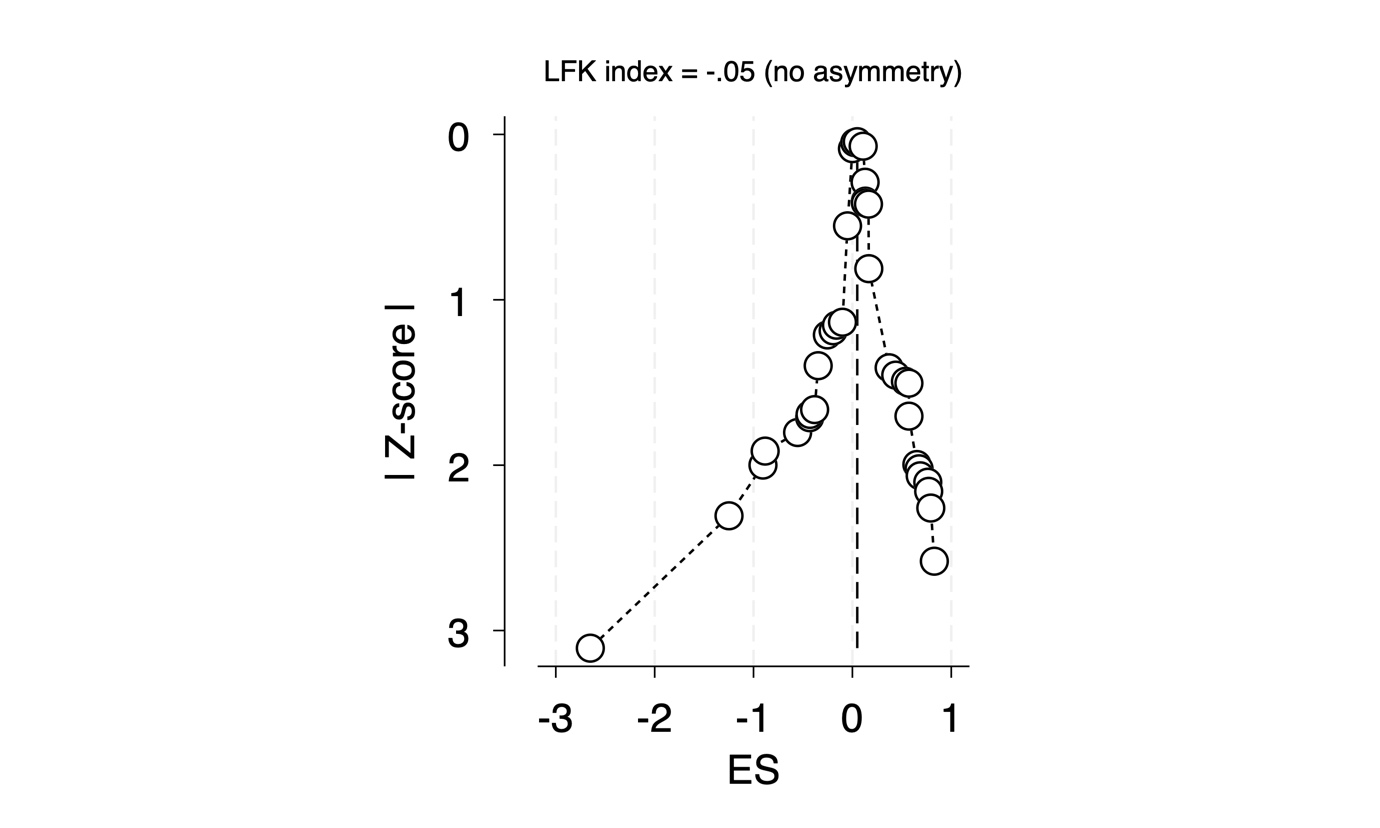

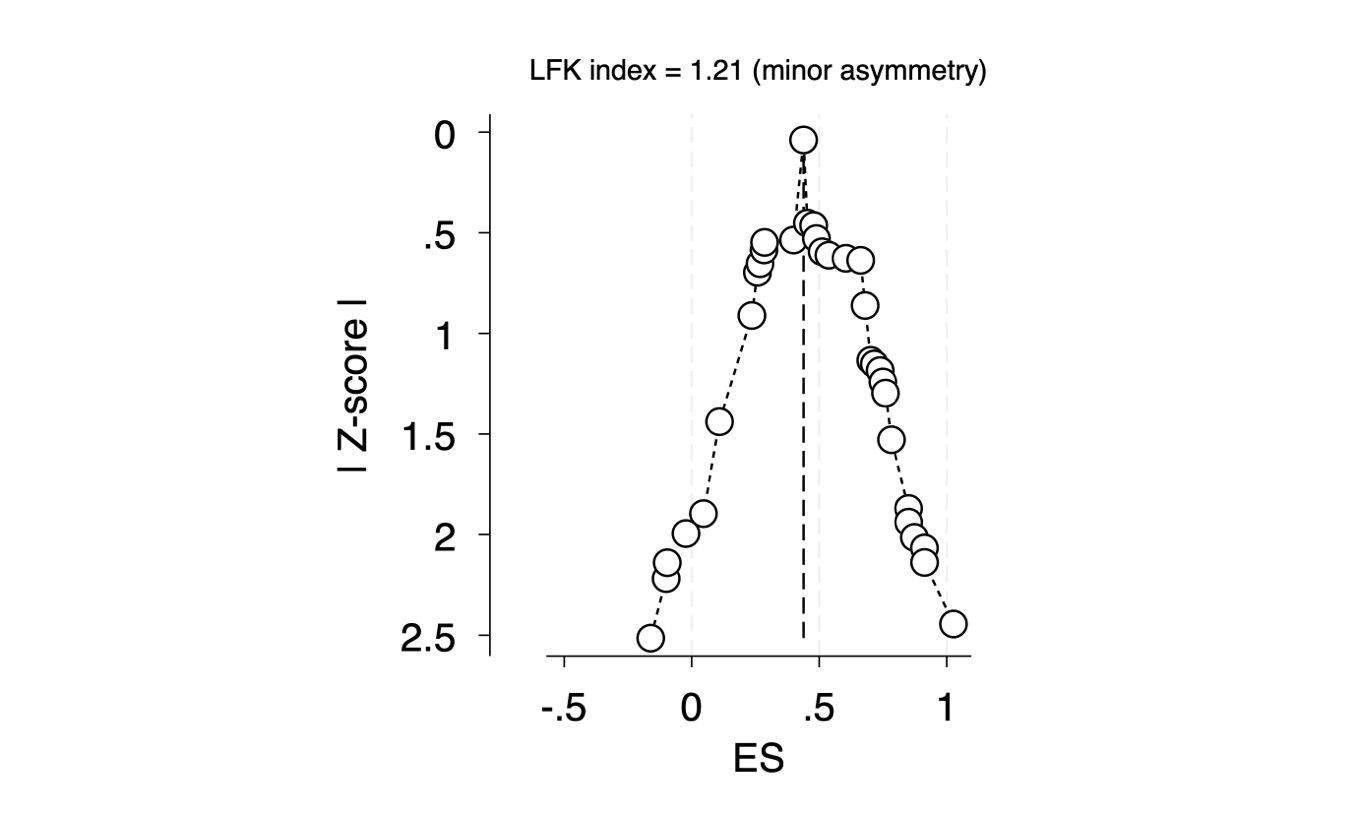

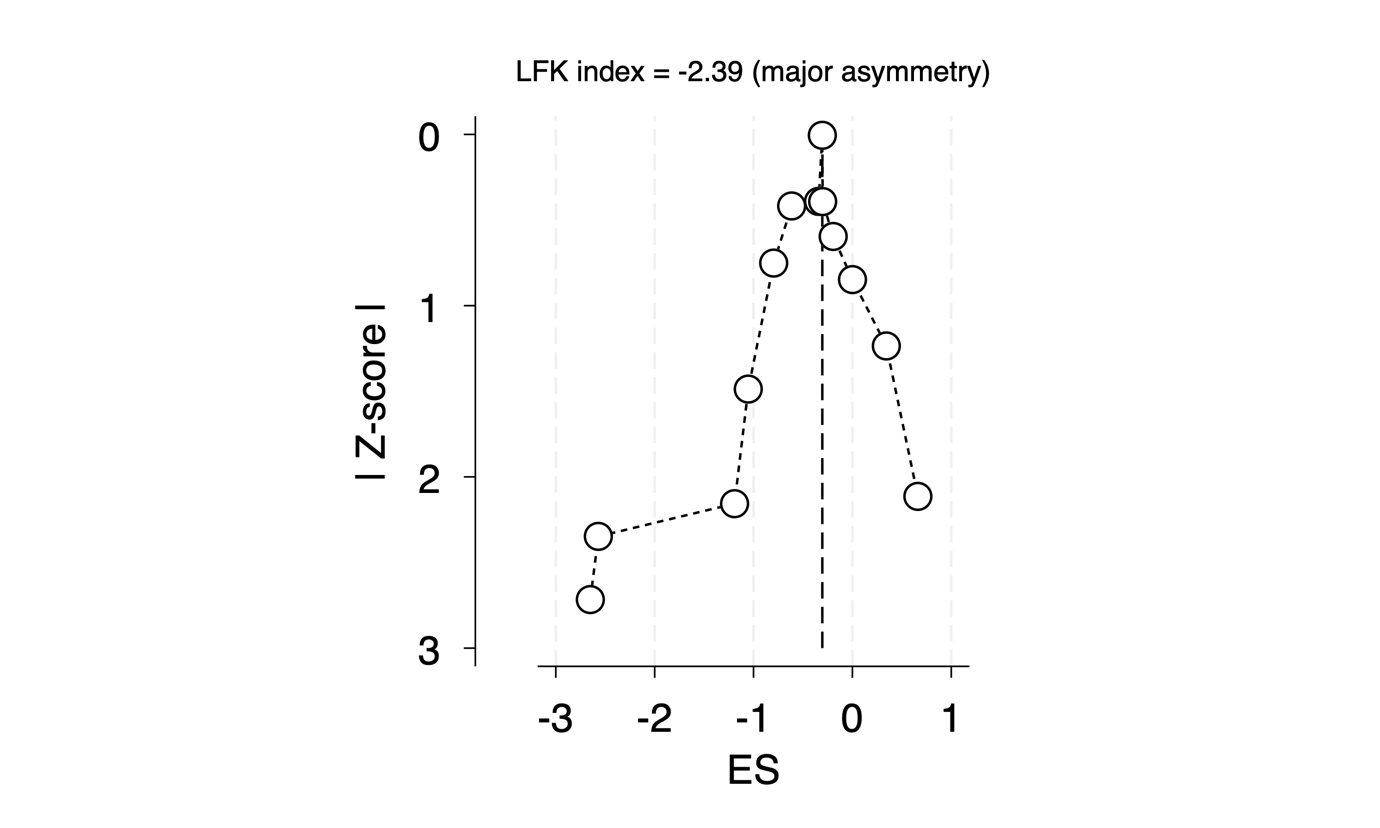

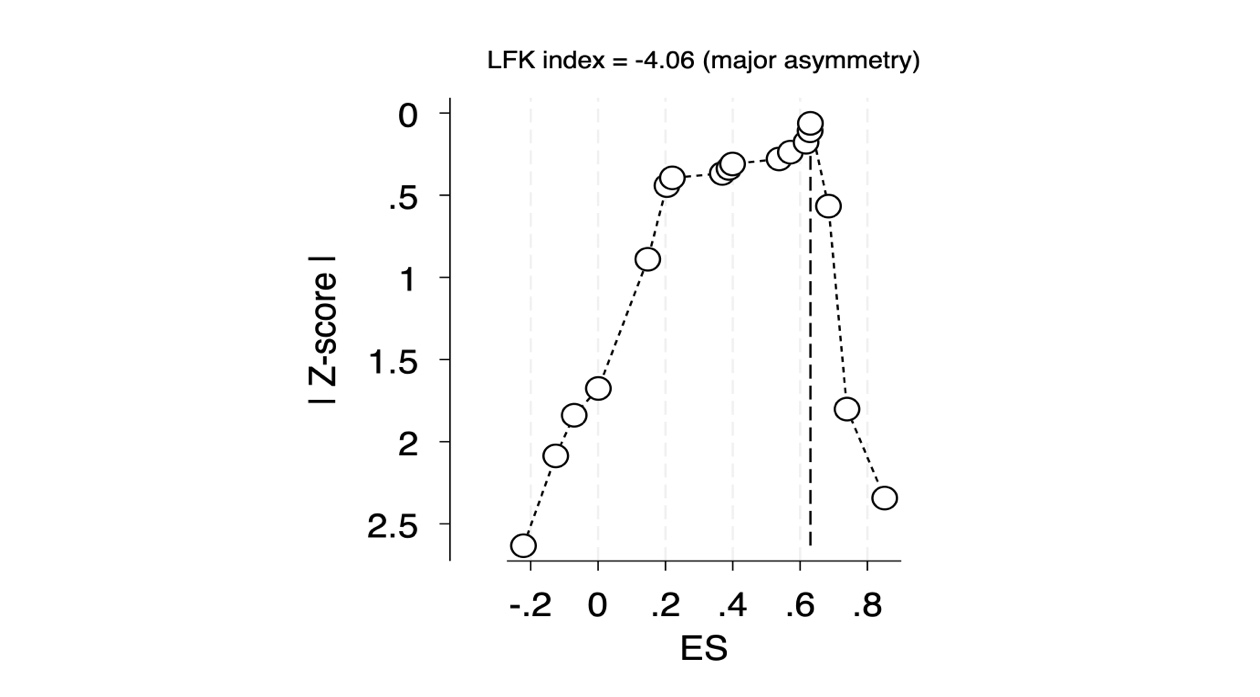
**

B

D

C

A
